# Bioisosteric Replacement of Amides with 1,2,3-Triazoles Improves Dopamine D4 Receptor Ligand Pharmacokinetics

**DOI:** 10.1021/acsptsci.5c00646

**Published:** 2026-01-09

**Authors:** Mohammad Alkhatib, Franziska M. Jakobs, John N. Hanson, Ashley N. Nilson, Amy E. Moritz, Tian Li, Afua B. Faibille, Lindsay A. Bourn, Peter A. Ramdhan, Joseph Ricchezza, Shannon Jordan, Diandra Panasis, Norman Nguyen, Nitish Kasarla, Bryant Wang, Sergio Sola Garcia, Julianna Saez, James Paule, Chae Bin Lee, Rana Rais, Barbara S. Slusher, David R. Sibley, Chenglong Li, Thomas M. Keck, Comfort A. Boateng

**Affiliations:** † Department of Basic Pharmaceutical Sciences, Fred Wilson School of Pharmacy, 465018 High Point University, One University Parkway, High Point, North Carolina 27268, United States; ‡ Department of Chemistry & Biochemistry, Department of Biological & Biomedical Sciences, College of Science and Mathematics, 3536 Rowan University, 201 Mullica Hill Road, Glassboro, New Jersey 08028, United States; § Department of Neurology, Johns Hopkins Drug Discovery, 1500 The Johns Hopkins University School of Medicine, 855 N. Wolfe Street, Baltimore, Maryland 21205, United States; ∥ Molecular Neuropharmacology Section, National Institute of Neurological Disorders and Stroke-Intramural Research Program, 2511 National Institutes of Health, Bethesda, Maryland 20892, United States; ⊥ Department of Medicinal Chemistry, University of Florida College of Pharmacy, 1345 Center Drive, Gainesville, Florida 32610, United States

**Keywords:** dopamine D_4_ receptor, agonist, antagonist, 1,2,3-triazole, bioisostere, pharmacokinetics

## Abstract

Dopamine D4 receptor (D_4_R) signaling affects decision-making, memory formation, cognition, and attention. Previously developed D_4_R-selective ligands were metabolically unstable *in vivo* due to amide bond linker hydrolysis. In this study, analog compounds were synthesized using click chemistry, bioisosterically replacing amides with a 1,2,3-triazole linker. Herein, we report 1,2,3-triazole analogs maintained high D_4_R affinity and subtype selectivity but had slightly reduced functional efficacy in cAMP and β-arrestin recruitment assays. Using rat and human liver microsomes to evaluate phase I metabolism, we determined that amide ligands were more metabolically unstable in rat microsomes, and the triazole substitutions enhanced compound stability. Four compounds were evaluated in rat pharmacokinetics studies. In particular, **17** (antagonist) and **18** (low-efficacy partial agonist) had desirable results in plasma half-life and brain exposure measures. These new analogs are suitable for behavioral studies in rats and represent improved molecular tools to further explore D_4_R signaling in rodent models.

## Graphical abstract

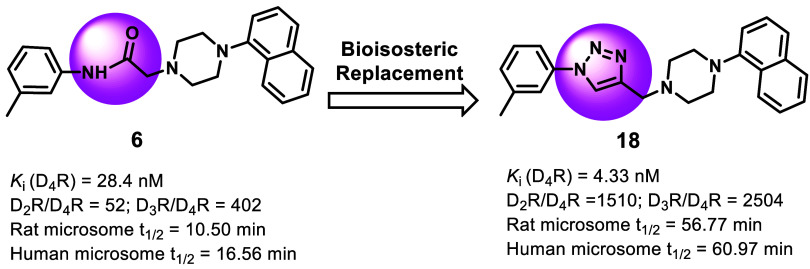

The catecholamine neurotransmitter dopamine (DA) signals by binding and activating dopamine receptors (DRs), a family of G protein-coupled receptors (GPCRs). DRs are subcategorized on the basis of signaling and sequence homology into the excitatory D_1_-like receptors, which includes dopamine D_1_ and D_5_ receptors (D_1_R, D_5_R), and the inhibitory D_2_-like receptors, which includes dopamine D_2_, D_3_, and D_4_ receptors (D_2_R, D_3_R, and D_4_R).^1^ All D_2_-like receptors share a similar signaling mechanism, coupling to Gα_i/o_ G proteins and recruiting β-arrestin.^2^ They also share substantial amino acid sequence homology in their orthosteric binding sites. However, they vary in their expression patterns within the brain and in their synaptic localization.^1,2^

D_4_Rs in the brain are mainly found in the hippocampal (HC) and prefrontal cortical (PFC) regions and have a lower overall level of expression compared to D_2_Rs and D_3_Rs, which are located primarily in the basal ganglia, striatum, and pituitary gland. Drugs targeting D_2_Rs and D_3_Rs can alter locomotor function and motivated states, and D_2_Rs are a primary target for antipsychotic drugs.^3,4^ In contrast, the activity of D_4_Rs located in the HC and the PFC influences exploratory behavior, attention, and performance in cognitive tasks, such as novel object recognition and inhibitory avoidance.^4–7^ Activating D_4_Rs could be a route for a potential treatment for cognitive deficits associated with attention-deficit/hyperactivity disorder (ADHD) and schizophrenia.^8–12^ Preclinical studies showed D_4_R agonists improved performance in cognitive tasks, such as novel object recognition tasks, 5-trial repeated acquisition inhibitory avoidance tasks, and social recognition tasks.^6,13,14^ Recent studies indicate that pharmacological activation of D_4_Rs could also minimize the negative effects of opioid drugs such as morphine.^15,16^ Antagonizing D_4_Rs might be helpful in treating l-DOPA-induced dyskinesias and substance use disorders (SUDs), particularly psychostimulant use disorders.^11,17–23^ A better understanding of D_4_R-mediated signaling is crucial for the development of novel pharmacotherapeutic treatments to treat these complex pathologies.

Despite the clinical significance, there are currently no FDA-approved medications for treating psychostimulant use disorders, nor are there FDA-approved medications that selectively target D_4_R. A recent resurgence in drug development targeting D_4_R^20,24^ has identified a range of new selective ligands, particularly piperidine- and piperazine-containing compounds,^25–28^ including some with antiglioblastoma effects.^29–31^

This study is part of a longitudinal effort by our group to create novel ligands with high D_4_R affinity and selectivity in order to investigate their effects in animal models of SUDs. In a previous study, the arylpiperidine A-412997 (**1**)—a D_4_R-selective, high-efficacy partial agonist—served as a template to develop a series of novel compounds using rational drug design, structure–activity relationship (SAR) analyses, and molecular dynamics (MD) simulations (Figure 1). This work resulted in a series with high D_4_R affinity, excellent selectivity over D_2_R and D_3_R, and a range of partial agonist and full antagonist efficacies.^32^ However, follow-up behavioral studies with lead compounds from this series suggested that there may have been pharmacokinetic limitations with these compounds. This was confirmed using *in vitro* pharmacokinetic studies that determined that the structural template was labile, with the amide linker consistently identified as the key site of both Phase I and non-Phase I metabolism (see metabolite identifications for **3**, **5**, and **6** in Figures S1 and S2, each showing dealkylated metabolic products with cleavage occurring at the amide linker).

**Figure 1:**
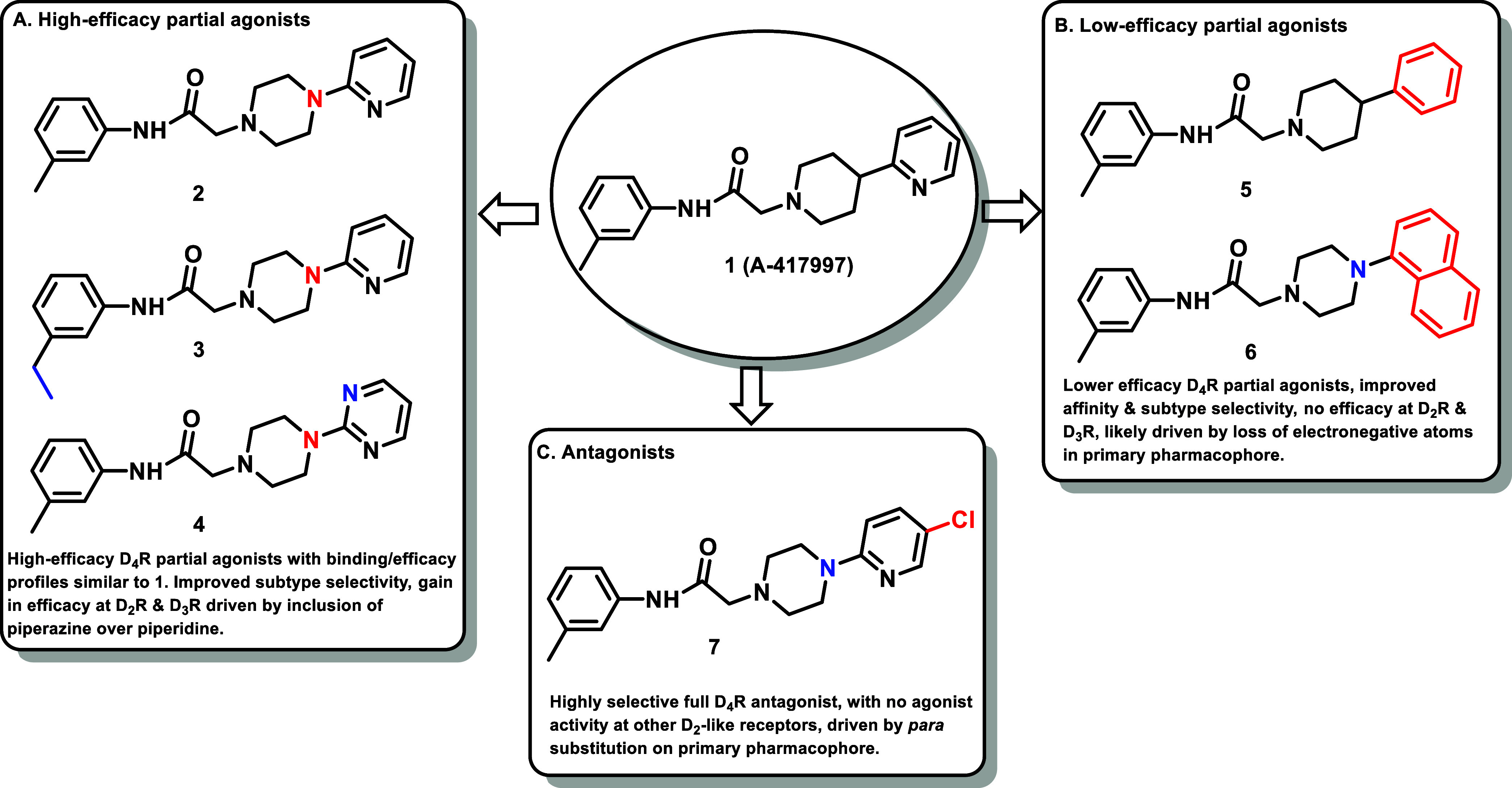
Three classes of modifications to the structure of **1** resulting in different binding and efficacy profiles at D_2_-like receptors.^32^ Structural differences from **1** are noted in blue, while changes driving observed pharmacodynamic shifts are shown in red. These six compounds served as the templates for new 1,2,3-triazole-containing analogs.

Herein, we report on the design and testing of a new analog library featuring a 1,2,3-triazole substitution of the amide linker in our previous library.^32^ The 1,2,3-triazole linker maintains many of the physicochemical properties of the amide (*e.g.*, size, rigidity, hydrogen bond acceptors and donors) and thus can be considered bioisosteric and would be predicted to minimally impact D_2_-like binding and efficacy.^33–36^ 1,2,3-triazoles should also be less susceptible to some forms of drug metabolism, including CYP450-mediated oxidation^37^ and hydrolysis *via* amidase enzymes. The goal of this study was 2-fold: (1) to determine whether 1,2,3-triazole substitution could improve the pharmacokinetic stability of previously developed compounds, and (2) measure whether the triazole substitution would impact pharmacodynamic properties of these ligands. We chose to develop 1,2,3-triazole analogs of compounds **2**–**7** as they represent highly D_4_R-selective ligands with a range of efficacies at D_4_R. To test this hypothesis, six 1,2,3-triazole analogs were synthesized and compared to their parent amide compounds in binding and functional studies, *in silico* docking and molecular dynamics simulations, and liver microsomal studies. Four compounds (**14**, **15**, **17**, and **18**) were fully evaluated for *in vivo* pharmacokinetics in rats. Overall, the successful use of simple and efficient click chemistry in the creation of these 1,2,3-triazole linkers opens new pathways for future library development.

## Chemistry

Ligands were synthesized as outlined in Scheme 1 using routine click chemistry reactions as previously reported.^38^ The triazoles **14–19** (Scheme 1) were prepared starting from commercially available tosylate (**8**), which was displaced using commercially available arylpiperazine or arylpiperidine amines to give acetylene-containing arylpiperazines or arylpiperidines (**9–13**). These acetylenes (**9–13**) were coupled to commercially available azides, formed *in situ*, which provided the desired triazoles compounds (**14–19**).

**Scheme 1:**
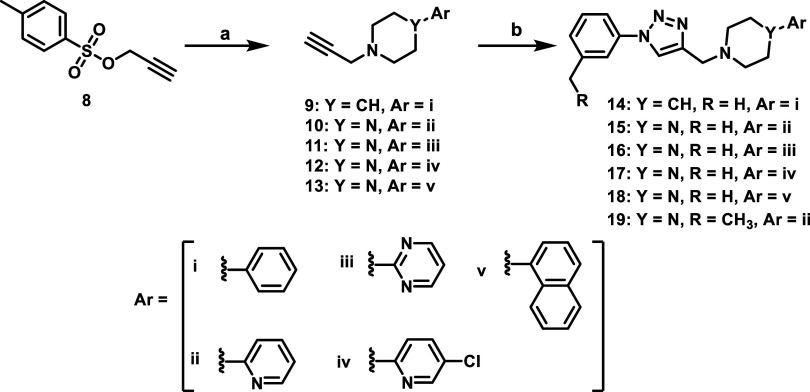
Scheme of 1,2,3-Triazole-Containing D_4_R-Selective Analogs^a^

a Reagents and Conditions: (a) K_2_CO_3_, NaI, appropriate arylpiperidine or arylpiperazine, acetone, reflux, 12 h; (b) (i) *t*-BuOH, H_2_O, copper­(II) sulfate pentahydrate, sodium ascorbate, appropriate azide, rt, 12 h.

## Pharmacological Results and Discussion

The primary objective of this study was to develop new D_4_R-selective ligands with improved pharmacokinetic profiles *via* bioisosteric replacement of the amide bond with 1,2,3-triazole-linked analogs. Compound **1** and several previously reported analogs (**2–7**) are shown in Figure 1.^32^ In order to obtain new analogs of compounds **2–7**, we employed click chemistry strategies by altering the amide linker creating 1,2,3-triazole-linked analogs with the goals of maintaining high D_4_R affinity and selectivity while improving the pharmacokinetic profile.

To begin, new 1,2,3-triazole-linked analogs were tested in radioligand competition binding assays to determine the effect of the alternate linker on D_2_R, D_3_R, and D_4_R binding affinity. Membranes from HEK293 cells stably expressing the D_2_R, D_3_R, or D_4_R were prepared and the ability of each analog to displace the radioligand [^3^H]*N*-methylspiperone was determined. The affinity was determined using the Cheng-Prusoff equation as described in the Methods and are shown in Table 1. In addition, *c* Log *P* values were calculated to provide measures of polarity (Table 1). Overall, the majority of the compounds exhibited *c* Log *P* values of less than 5 and new triazole library members consistently demonstrated higher binding affinity for D_4_R over D_2_R and D_3_R.

**Table 1:**
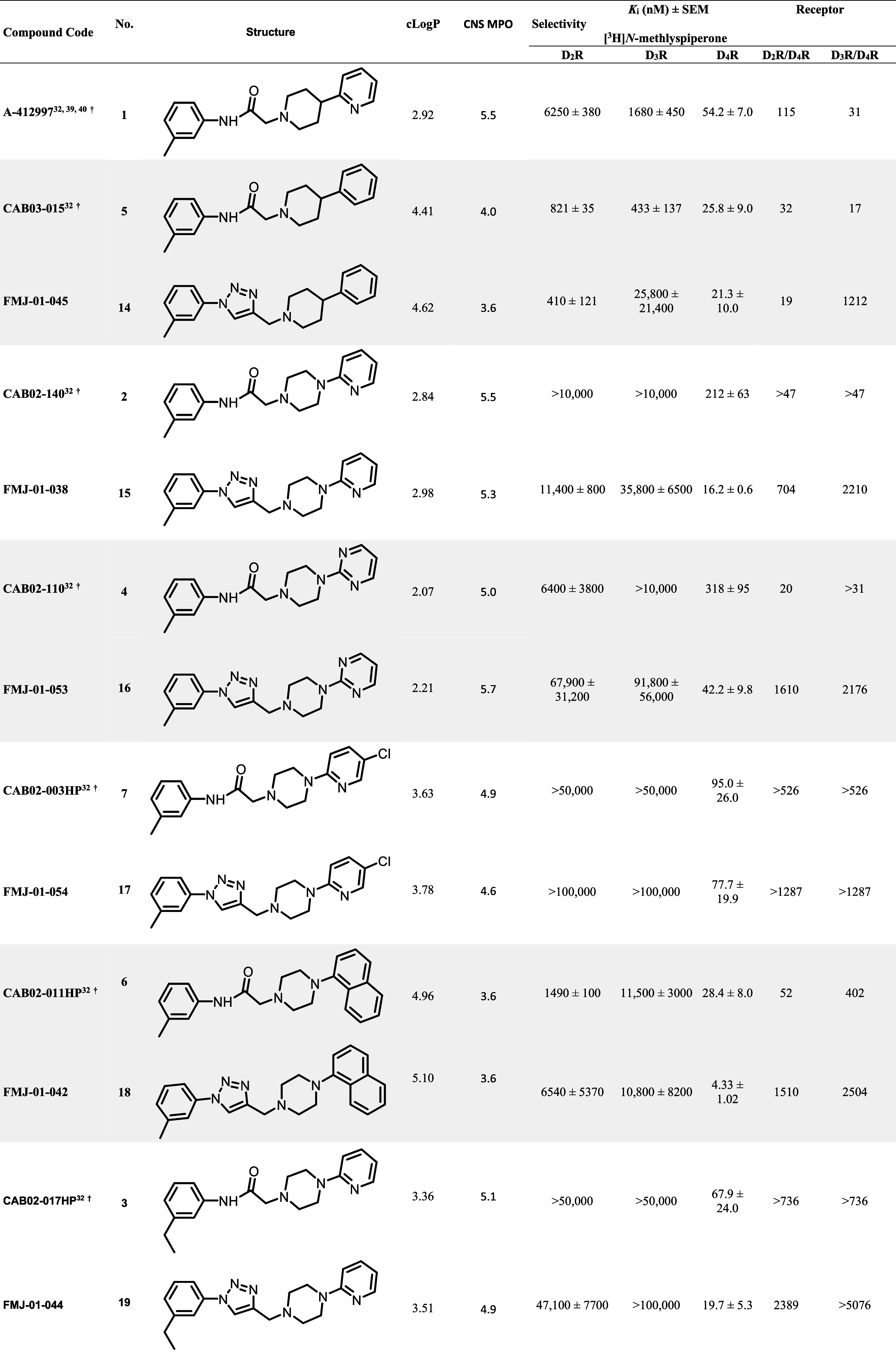
Human Dopamine D_2_-like Receptor Binding Data in HEK293 Cells for Ligands with Amide or 1,2,3-Triazoles Moieties^a^ ^,^ ^39,40^

a *K*_i_ values determined by competitive inhibition of [^3^H]*N*-methylspiperone binding in membranes harvested from HEK293 cells stably expressing hD_2_R, hD_3_R, or hD_4_R. All *K*_i_ values are presented as means ± SEM.

† Data previously reported in Keck and Free et al.^32^ CNS Multiparameter Optimization (MPO) scores were calculated using ChemDraw (version 23.0) and Chemaxon’s CNS MPO Score Predictor in Marvin.^41,42^

Comparing the binding affinities across each pair of amide and triazole analogs, all triazole analogs had comparable or improved affinity for the D_4_R compared to their amide analogs (Table 1), indicating that the substitution is well-tolerated. **14** maintained binding affinity for D_4_R (21.3 nM) comparable to its analog **5** (25.8 nM), with 19-fold and 1212-fold selectivity over D_2_R and D_3_R, respectively. **15** displayed higher binding affinity for D_4_R (16.2 nM) compared to its analog **2** (212 nM), resulting in improved 704-fold and 2,210-fold selectivity over D_2_R and D_3_R, respectively. **16** displayed higher binding affinity for D_4_R (42.2 nM) compared to its analog **4** (318 nM), resulting in improved 1,610-fold and 2,176-fold selectivity over D_2_R and D_3_R, respectively. **17** displayed higher binding affinity for D_4_R (77.7 nM) comparable to its analog **7** (95.0 nM), with >1287-fold selectivity over D_2_R and D_3_R. **18** displayed higher binding affinity for D_4_R (4.33 nM) comparable to its analog **6** (28.4 nM), with 1,510-fold and 2504-fold selectivity over D_2_R and D_3_R, respectively. **19** displayed higher binding affinity for D_4_R (19.7 nM) comparable to its analog **3** (67.9 nM), with 2389-fold and >5076-fold selectivity over D_2_R and D_3_R, respectively.

While the triazole substitution typically resulted in modestly favorable affinity gains at D_4_R, we do not want to overinterpret the comparison of new binding results with our prior literature reports. Therefore, a more conservative evaluation of these results indicates that the triazole substitution shows no negative impact on D_4_R affinity or subtype selectivity.

We investigated the effects of the triazole linker on β-arrestin recruitment to D_2_-like receptors. Functional analyses of each compound were completed using the DiscoverX β-arrestin recruitment assay (Table 2). Analogs were tested in both agonist and antagonist modes using Chinese hamster ovary (CHO) cells stably expressing a prolink-tagged D_2_R, D_3_R, or D_4_R and a β-arrestin2 tagged with the remaining portion of β-galactosidase in an enzyme complementation assay. In agonist mode, compounds were tested alone and *E*_max_ values for each compound are in comparison to DA. In antagonist mode, compounds were tested in the presence of an EC_80_ concentration of DA (1 μM) and all assays were normalized to spiperone. In general, the triazole analogs displayed potencies and efficacies consistent with their respective amide analogs. The triazole substitutions had minimal impact on the potencies of the compounds for the D_2_-like receptors with a few exceptions detailed below. At the D_4_R, triazole **14** was less potent (1200 nM) than the amide analog **5** (135 nM) but the efficacy was not affected (93%). The efficacies indicated they were antagonists but had very low potency (>6000 nM) at the D_2_R and D_3_R. The triazole **16** did not show partial agonist activity at the D_4_R while the amide **4** analog had 25% efficacy and 278 nM potency for recruiting β-arrestin. There was a similar effect with **6** and **18** as well as **3** and **19** pairs of amide *vs* triazole. Both amides show low partial agonist activity while the triazole analogs did not. All the D_2_R, D_3_R, and D_4_R β-arrestin recruitment results are shown in Table 2 and indicate that the amide substitution with the triazole was well-tolerated and was not detrimental for β-arrestin recruitment antagonism, with the exception of **14**. Taken together, these binding and functional results indicate that the triazole linker was well-tolerated and even improved D_4_R affinity and subtype selectivity for many of the analogs tested.

**Table 2:** D_2_R-, D_3_R-, and D_4_R-Mediated β-Arrestin Recruitment^a^,^c^,^d^

	D_2_R				D_3_R				D_4_R				EC_50_		IC_50_	
compound	*E*_max_ (%)	EC_50_ (nM)	Ant. (%)	IC_50_ (nM)	*E*_max_ (%)	EC_50_ (nM)	Ant. (%)	IC_50_ (nM)	*E*_max_ (%)	EC_50_ (nM)	Ant. (%)	IC_50_ (nM)	D_2_R/D_4_R	D_3_R/D_4_R	D_2_R/D_4_R	D_3_R/D_4_R
Dopamine	99.3 ± 0.7	16.0 ± 3.4			99.3 ± 0.4	4.9 ± 0.6			90.0 ± 0.7	300 ± 53			0.5	0.04		
Spiperone			100 ± 0	0.36 ± 0.05			98.7 ± 1.0	2.4 ± 0.4			100 ± 0	1.0 ± 0.3			0.36	2.4
**1** ^b^	NA	NA	94.8 ± 2.8	5850 ± 1800	ND	>100,000	ND	>100,000	22.5 ± 4.0	473 ± 457	81.7 ± 2.7	191 ± 98		>4	31	>524
**5** ^b^	NA	NA	99.7 ± 0.3	7690 ± 2300	NA	NA	ND	>50,000	14 ± 0.3	242 ± 89	93.3 ± 1.8	135 ± 65			57	>371
**14**	NA	NA	112 ± 3	6090 ± 1340	NA	NA	81.6 ± 0.3	6300 ± 2100	ND (12% at 100 μM)	>100,000	93.3 ± 5.9	1200 ± 160			5.1	5.3
**2** ^b^	23.9 ± 5.1	26,200 ± 12,400	76.5 ± 6.9	16,000 ± 5200	49.4 ± 2.0	6350 ± 2,620	ND	>100,000	30.7 ± 6.4	394 ± 294	78.9 ± 3.1	313 ± 215	66	>16	51	>320
**15**	16.5 ± 9.8	>100,000	87.7 ± 2.6	9050 ± 2500	110 ± 29	3600 ± 480	ND	ND	ND (14% at 100 μM)	>100,000	82.8 ± 3.3	310 ± 49			29	
**4** ^b^	39.6 ± 5.3	8320 ± 3460	78.9 ± 8.6	25,000 ± 5000	58.4 ± 6.6	5,580 ± 1610	ND	>100,000	24.7 ± 5	278 ± 167	80.6 ± 3.0	197 ± 115	30	>20	127	>509
**16**	25.3 ± 4.9	6870 ± 2500	73.2 ± 4.6	19,000 ± 3700	75 ± 14	>100,000	105 ± 4	>10,000	ND (14% at 100 μM)	>100,000	85.2 ± 2.4	140 ± 35			136	71
**7** ^b^	NA	NA	100 ± 0	\>100,000	NA	NA	ND	ND	NA	NA	100 ± 0	7780 ± 2170			>13	
**17**	NA	NA	ND	ND	NA	NA	ND	ND	NA	NA	91.6 ± 4.7	3800 ± 720				
**6** ^b^	NA	NA	100 ± 0	>100,000	NA	NA	ND	ND	16.4 ± 3.9	9210 ± 6240	96.7 ± 2.7	4250 ± 1080			>24	
**18**	NA	NA	87.8 ± 26.7	4700 ± 1100	NA	NA	ND	ND	ND (8% at 10 μM)	>10,000	92.1 ± 12.1	3600 ± 1100			1.3	
**3** ^b^	18.7 ± 0.4	3890 ± 1880	100 ± 0	88,400 ± 11,600	44.7 ± 5.9	2760 ± 470	100 ± 0	88,200 ± 9600	26.2 ± 5.1	133 ± 60	59.7 ± 4.6	370 ± 105	29	21	239	238
**19**	ND	ND	102 ± 2	28,300 ± 10,500	ND (35% at 100 μM)	>100,000	ND	ND	ND (13% at 100 μM)	>100,000	92.9 ± 4.6	420 ± 120			67	

a Efficacy/antagonist % (Ant. %) values obtained from nonlinear regression of meaned data obtained from at least three independent experiments with triplicate measures. Values are presented as means ± SEM.

b Data previously reported in Keck and Free et al.^32^

c Dopamine was used as a control in all agonist mode assays. Spiperone was included in all antagonist mode assays for the D_2_R and D_4_R. -: not tested. NA: No Activity. ND: Not Determined due to incomplete curves that did not saturate; for ND *E*_max_ values, the average activity at the highest tested dose is provided as (% activity at dose in μM).

d Compounds were tested alone (agonist mode) and with an EC_80_ concentration of dopamine (antagonist mode) for their ability to alter β-arrestin recruitment to hD_2_R, hD_3_R, and hD_4_R[Table-fn t2fn1].

We investigated the effects of the triazole linker on D_4_R-mediated inhibition of forskolin-stimulated cAMP accumulation. Functional analyses of each compound were completed using the LANCE cAMP assay (Table 3). All compounds were tested in both agonist and antagonist modes using CHO-K1 cells stably expressing the human D_4_R. In agonist mode, all compounds were tested in the presence of 10 μM forskolin and *E*_max_ values for each compound are in comparison to DA. In antagonist mode, compounds were tested in the presence of 10 μM forskolin and in the presence of an EC_80_ concentration of DA (10 nM) and all assays were normalized to spiperone. Efficacy and potency values for **2**-**7** are very similar to those previously reported,^32^ with the exception of **6**—this compound has poor aqueous solubility, which can impact its activity in binding and functional studies that use different buffer conditions. Generally, triazole analogs displayed potencies consistent with their respective amide analogs, but with a trend toward modestly reduced intrinsic efficacy and a corresponding increase in antagonist efficacy, indicating that the triazole substitution does reduce receptor activation. Notably, *E*_max_ values in this cAMP accumulation assay are considerably higher than those in the β-arrestin recruitment assay above, and potencies are considerably higher. This is consistent with our prior findings and likely results from differences in receptor reserves and signaling capacities across very different assay readouts: the effects on cAMP accumulation as part of a signaling cascade amplifies drug effects whereas the β-arrestin recruitment assay is unamplified.

**Table 3:** D_4_R-Mediated Effects on cAMP Synthesis^a^,^b^

	D_4_R			
compound	*E*_max_ (%)	EC_50_ (nM)	Ant. (%)	IC_50_ (nM)
Dopamine	100 ± 0	2.76 ± 0.96		
Spiperone			100 ± 0	14.6 ± 1.45
**5**	42 ± 8	7.54 ± 2.21	47 ± 1	42.4 ± 10.6
**14**	30 ± 5	44.5 ± 17.4	63 ± 2	84.6 ± 15.6
**2**	65 ± 3	2.92 ± 0.15	16 ± 1	ND
**15**	43 ± 6	5.29 ± 1.39	40 ± 1	57.6 ± 5.44
**4**	60 ± 4	7.93 ± 2.12	29 ± 5	ND
**16**	39 ± 7	7.68 ± 1.88	45 ± 3	67.9 ± 11.3
**7**	NA	NA	110 ± 7	2870 ± 817
**17**	NA	NA	85 ± 1	134 ± 36.4
**6**	ND (16% at 33 μM)	ND	91 ± 7	>50,000
**18**	24 ± 7	2140 ± 1065	72 ± 5	904 ± 102
**3**	59 ± 4	5.38 ± 1.08	30 ± 1	267 ± 115
**19**	41 ± 7	9.00 ± 2.48	47 ± 1	41.9 ± 1.32

a Efficacy/antagonist % (Ant. %) values obtained from nonlinear regression of meaned data obtained from at least three independent experiments with triplicate measures. Values are presented as means ± SEM. Agonist mode was run in the presence of 10 μM forskolin. Antagonist mode was run in the presence of 10 μM forskolin and 10 nM dopamine. -: not tested. NA: No Activity. ND: Not Determined due to incomplete curves that did not saturate; for ND *E*_max_ values, the average activity at the highest tested dose is provided as (% activity at dose in μM).

b Compounds were tested alone (agonist mode) and with an EC_80_ concentration of dopamine (antagonist mode) for their ability to alter cAMP accumulation at hD_4_R.

Taken together, these binding and functional results indicate that the triazole linker was generally well-tolerated, maintaining D_4_R affinity, subtype selectivity, and overall activity profiles compared to their amide analogs.

### *In Silico* Studies of Compounds **2–7** and **14–19**

Overall, we found a modest but consistent improvement in D_4_R affinity in the 1,2,3-triazole analogs compared to their amide counterparts. To determine the mechanisms of these improvements, we used molecular dynamics (MD) simulations of the structure of the human D_4_R in complex with nemonapride (PDB ID: 5WIU)^43^ in complex with l-dopamine to create a model of D_4_R in an agonist-bound state. Following MD simulations, compounds **2–7** and **14–19** were docked into the receptor’s orthosteric site. Models with the highest docking score for each receptor–ligand pair were then analyzed using DeepAtom^44^ to predict the binding energies of each compound (Table 4).

**Table 4:** Deep Atom Binding Affinity Scores for Compounds **2-7** and **14-19** at the D_4_R^a^

deep atom binding affinity scores for amide analogs		deep atom binding affinity scores for triazole analogs	
compound number	score (kcal/mol)	compound number	score (kcal/mol)
**5A** ^b^	–10.16	**14A** ^b^	–10.45
**5B** ^b^	–10.10	**14B** ^b^	–10.59
**2**	–9.61	**15**	–10.48
**4**	–9.63	**16**	–10.49
**7**	–9.63	**17**	–10.38
**6**	–9.01	**18**	–10.19
**3**	–9.56	**19**	–10.45

a The above Table 4 displays calculated binding energy scores using DeepAtom.^44^ The left columns represent amide compounds, with matching triazole-based analogs in the right columns.

b Compounds **5A** and **5B** represent probable alternative docking pose conformations of amide **5**. Similarly, compounds **14A** and **14B** represent probable alternative docking pose conformations of triazole analog **14**. The **A** conformations represent the “opposite pose”, and the **B** conformation represent the “consistent pose” (*i.e.*, conformationally consistent with the docking of **2–7** and **14–19**).

After docking, 15 poses were generated for each compound. Figure S12 shows the surface of the D_4_R from afar and a zoom-in of the binding site, composed of the orthosteric and extended-binding pocket (EBP) sites. All ligands showed consistency in binding mode and orientation, however analogs **5** and **14** showed a matching variant “opposite pose” described in more detail below. A representative set of amides and triazole compounds were chosen (**2** and **15**, respectively) to illustrate comparative binding interactions. Figure 3 illustrates the interactions of **2** and **15** with the amino acid side chains found in the binding site.

The poses seen consistently among all compounds, exemplified by amide **2** and triazole **15**, share key features. The methyl phenyl group of these compounds prefer placement into the EBP, which is formed through W101. It appears that this pocket cannot hold large aromatic or hydrophobic moieties. In Figure 2E, D115 displayed a salt bridge with the protonated tertiary amine of **15**, a conserved interaction among dopamine receptor binders. Compound **2** shows the same salt bridge formation, but the amide nitrogen provides an additional interaction in the form of a hydrogen bond with D115.

**Figure 2:**
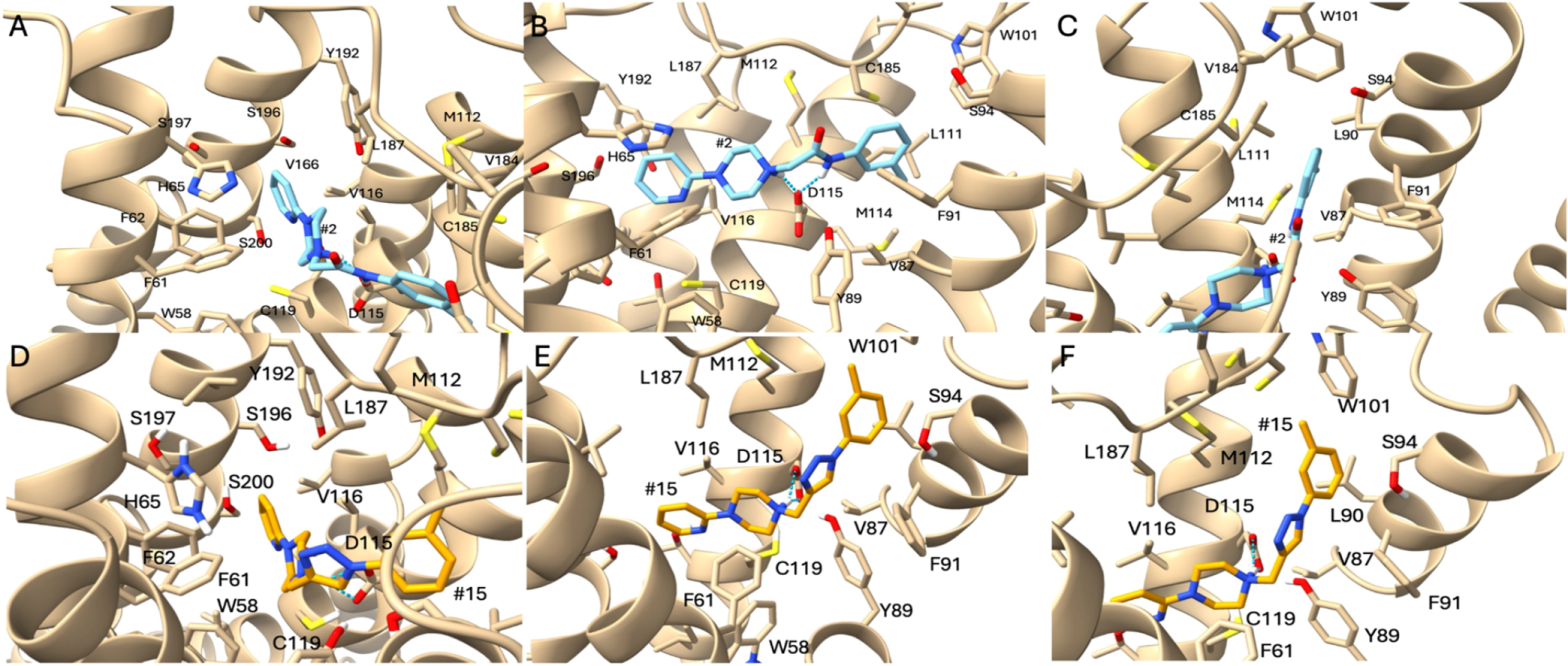
(A–C) Compound **2** in complex with D_4_R. Panels A and C display interactions in the OBP and EBP, respectively. (D–F) Compound **15** in complex with D_4_R. Panels D and F display interactions in the OBP and EBP, respectively.

Figure 2A,D display interactions within the orthosteric binding pocket (OBP) for **2** and **15**, respectively. In this pocket, hydrophobic interactions dominate. Normally with endogenous dopamine, the hydroxyls of the catechol would interact with S196/197, however, these compounds do not have this ability and thus will not form those interactions. Pi-pi interactions can be seen through the ring and F61/62, with slight aromatic interactions of H65 and hydrophobic interactions from V116/166, L187, and C119.

Figure 2C,F show the compounds forming interactions within the extended-binding pocket (EBP). Hydrophobic and pi-pi interactions also dominate here. The compounds form hydrophobic interactions with M114, V87, L90, L111, and V184. A nearby F91 could be used for potential pi-pi interactions with the triazole-based compounds through the triazole ring.

As mentioned previously, compounds **5** and **14** showed two plausible orientations while docking to D_4_R, a “consistent pose” (*i.e.*, conformationally consistent with the docking of **2–7** and **14–19**) that maintains the interactions described above, as well as an “opposite pose” with a flipped orientation. The Maestro docking functionality gave equivalent docking scores to the “opposite pose” and “consistent pose” orientations. After using DeepAtom, both pose orientations produce similar binding energy values (Table 4). Figure S13 displays triazole-based compound **14** in the “opposite pose” (panel A; **14A** in Table 3) and “consistent pose” (panel B; **14B** in Table 3). Surprisingly, it appears that the phenyl ring on compounds **5** and **14** can be equally accommodated by either side of the binding site.

Figure S14 displays amide-based compound **5** in the “opposite pose” (panel A; **5A** in Table 4) and “consistent pose” (panel B; **5B** in Table 4). In these images the amide nitrogen is no longer participating in H-bonding with the conserved D115; we are unsure why no docking poses for **5** showed this interaction while all other compounds did. As with the alternate poses for compound **14**, it is possible that the similarly sized aromatic rings on each end of the ligand can be accommodated by either end of the binding site.

Considering the overall docking results, the “consistent pose” was strongly preferred when the aromatic ring of **5** or **14** (a methylphenyl) features moieties that create a more electron-deficient ring, such as pyridines or chlorine. The possibility of sterics being a player here may contribute to equal favoring of either pose. The accommodability of the binding site could also be impacted by the use of a select number of frames during the MD simulations performed—it may be possible that throughout the trajectory, one pose may be preferred over the other. The contribution of electron-withdrawing groups and electron-donating groups may additionally play a role within the orthosteric site. There are more aromatic moieties in the OBP compared to the EBP.

All docking poses underwent binding affinity calculations using DeepAtom, a 3D-convolutional neural network used to calculate binding affinities with high accuracy (Table 4). Overall, the triazole-based compounds produced a more negative binding energy compared to the amide-based compounds. This appears to correspond with the modest improvement in D_4_R affinity seen in the radioligand binding studies presented above.

### *In Vitro* Metabolic Stability Studies of Compounds **2–7** and **14–19**

We evaluated the Phase I metabolic stability of compounds **2–7** and **14–19** using rat and human liver microsomes, as previously described.^45^ Incubation of compounds **2–7** and **14–19** with rat (Figure 3) or human (Figure 4) liver microsomes in the presence of NADPH resulted in time-dependent degradation. Overall, these results clearly indicate that amides **2–7** have lower metabolic stability compared to matching triazoles **14–19** in rat liver microsomes. Considering the main goal of this study was to identify a mechanism to improve compound stability in rats for further behavioral studies, this proved to be a successful substitution. Amides **2–7** had greater overall stability in human liver microsomes, and the triazole substitution resulted in a mix of improved, unchanged, and reduced microsomal half-life calculations, ranging from approximately 37–64 min, which are still suitable for continued development. HPLC traces of **2–7** and **14–19** and the major metabolite of **2–7 (**hydrolyzed amide product) are shown in Figures S1–S5.

**Figure 3:**
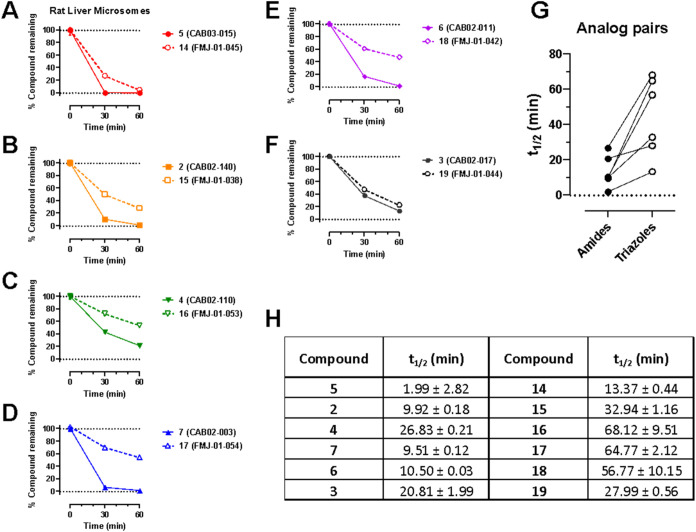
Phase I metabolic stability of **2–7** and **14–19** in rat liver microsomes. (A–F): Data are presented as percent compound remaining (means ± SEM) at 0-, 30-, and 60 min following incubation with rat liver microsomes in the presence of NADPH. (G): pairwise comparison of calculated compound half-lives for each amide-triazole analog pair. (H): calculated half-lives for each compound, expressed as means ± SD, *n* = 3.

**Figure 4:**
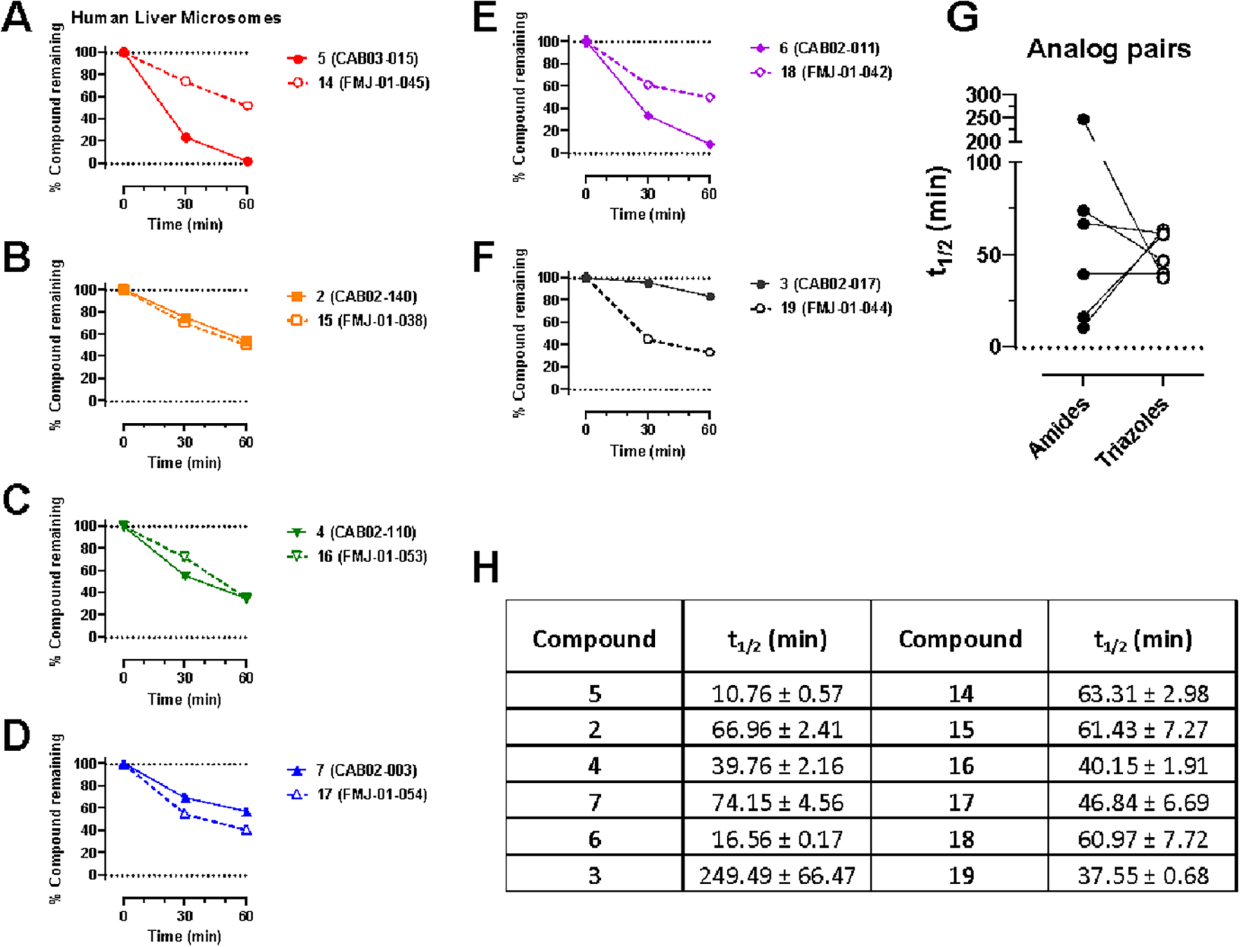
Phase I metabolic stability of **2–7** and **14–19** in human liver microsomes. (A–F): Data are presented as percent compound remaining (means ± SEM) at 0-, 30-, and 60 min following incubation with human liver microsomes in the presence of NADPH. (G): Pairwise comparison of calculated compound half-lives for each amide-triazole analog pair. (H) Calculated half-lives for each compound, expressed as means ± SD, *n* = 3.

We also evaluated the non-Phase I metabolic stability of compounds **2–7** and **14–19** using rat and human liver microsomes. Incubation of compounds **2–7** and **14–19** with rat (Figure 5A,B) and human (Figure 5C,D) liver microsomes in the absence of NADPH generally resulted in time-dependent compound degradation at a much slower rate than in the presence of NADPH. Notably, several amide compounds (**2–7**) have considerable microsomal instability—particularly in rat microsomal studies—even in the absence of the NADPH cofactor necessary for cytochrome P450-mediated metabolism. This may represent metabolism by hydrolases that can specifically attack the amide. Evidence in support of this hypothesis is shown by the remarkable stability of all triazole analogs (**14–19**) in the absence of NADPH in Figure 5B,D.

**Figure 5:**
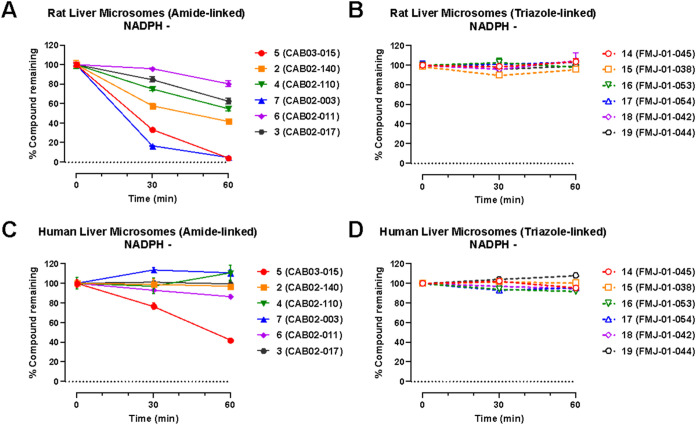
Non-Phase I metabolic stability of **2–7** and **14–19** in rat (A, B) and human (C, D) liver microsomes. Data are presented as percent compound remaining (means ± SEM) at 0-, 30-, and 60 min following incubation with rat or human liver microsomes in the absence of NADPH.

Evaluating these results across species, non-Phase I metabolism is considerably lower for the amides in human liver microsomes (Figure 5C) compared to rat liver microsomes (Figure 5A), likely highlighting a key species difference that drives some effects of the triazole substitution. While our data do not indicate the particular drivers of non-Phase I metabolism for these compounds, amidase-mediated hydrolysis can vary substantially across species, with activity levels strongly dependent on the specific substrates and enzyme isoforms involved. For example, some studies show higher amide deacetylase (AADAC) expression and activity in humans than in rats^46^ while other studies have shown the opposite trend for different amide scaffolds.^47^ Thus, although NADPH-free conditions largely rule out CYP450-mediated oxidation, they do not exclude hydrolytic turnover by amidases or other nonoxidative enzymes whose abundance and specificity differ markedly between species. The greater turnover observed in rat microsomes under NADPH-free conditions could therefore reflect higher intrinsic activity of one or more hydrolase classes toward these particular amides.

The stability gains of **14** *versus* **5** in human liver microsomes appears heavily impacted by reduced non-Phase I metabolism. In contrast, the gains seen with **18** *versus* **6** in human liver microsomes likely involves more protection against Phase I metabolism as there was relatively little non-Phase I metabolism of **6**. In human liver microsomes, **19** *versus* **3** presents the largest divergence from our overall trend: metabolism of **3** in the presence of NADPH is quite slow and the inclusion of the triazole substitution in **19** surprisingly resulted in greater Phase I metabolism (Figure 4F). Since the metabolism of **3** is nonexistent in the absence of NADPH (Figure 5C), there were no gains to be had *via* this protection mechanism, thus this effect must be driven by the introduction of new NADPH-dependent metabolic routes in human liver microsomes. Overall, the triazole-containing set have calculated half-lives that are more similar across species compared to the amide parent compounds. This improvement in cross-species predictiveness of pharmacokinetics may prove useful for the further development of this class of compounds.

Compound lipophilicity can impact a range of ADME values, with higher lipophilicity (as measured by log *P*) associated with increased metabolic clearance. This is driven primarily by the fact that lipophilic compounds tend to have greater affinity for metabolic enzymes such as CYP450s.^48^ However, in our case, compounds **14** and **18** have longer half-lives in human liver microsomes compared to their less lipophilic amide counterparts (**5** and **6**), suggesting that while lipophilicity can influence metabolic behavior, other factors, including steric effects and electronic properties, may counterbalance the typical trend.

### Pharmacokinetic Assessment of **14, 15, 17**, and **18** in Rats

Given their adequate *in vitro* stability profiles, we next evaluated the *in vivo* pharmacokinetic profiles of **14**, **15**, **17**, and **18** in rats. Sprague–Dawley rats were dosed with 5 mg/kg (**14**, **15**, **17**) or 10 mg/kg (**18**), i.p., and plasma and brain levels of each drug were measured 0–6 h postdose. The results from the pharmacokinetic analyses are shown in Figure 6A–D.

**Figure 6:**
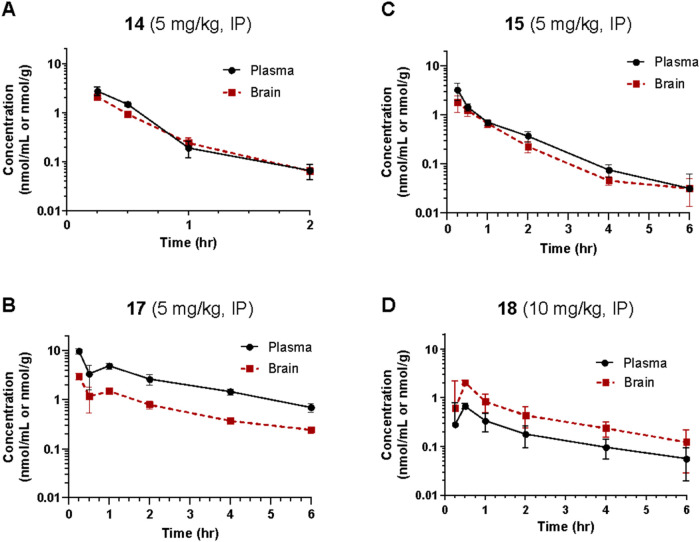
Time-dependent *in vivo* pharmacokinetic analyses of (A) **14**, (B) **15**, (C) **17**, and (D) **18** in Sprague–Dawley rats following intraperitoneal (i.p.) administration of 5 or 10 mg/kg drug. Data are presented as means ± SEM, *n* = 3 for each time point. The calculated pharmacokinetic parameters of each compound are provided in Table 5.

The calculated pharmacokinetic parameters of each compound are provided in Table 5. The intraperitoneal doses tested would each provide adequate brain exposure for possible behavioral studies with predicted brain concentrations that exceed *in vitro* IC_50_ and *K_i_* values for each compound. While compound **14** had the shortest half-life (≤24 min), with a brain *C*_max_ of 2.89 nmol/g, peak *in vivo* brain concentrations are expected to be greater than 2.8 μM. Compound **15** had a longer half-life (>66 min) with peak *in vivo* brain concentrations expected to be greater than 1.8 μM. Despite having the poorest brain penetration index, compound **17** (half-life ≥ 126 min) had the highest brain *C*_max_ and AUC, with peak *in vivo* brain concentrations expected to be greater than 3.0 μM. Compound **18** had the best brain penetration index (AUC_brain/plasma_ ratio > 2.6) and the longest half-life (∼2.5 h) of these compounds, with peak *in vivo* brain concentrations expected to be greater than 2.0 μM. The differences in brain penetration seen across these compounds may arise from several factors, such as different levels of plasma protein binding and the possibility that some of these compounds serve as substrates for P-gp efflux transport, which can correlate with compound lipophilicity.^49^

**Table 5:** Pharmacokinetic Parameters of **14**, **15**, **17**, and **18** in Rats

treatment	dose (mg/kg)	route	tissue	*C*_max_ (nmol/mL or nmol/g)	*T*_max_ (h)	AUC (nmol/mL·h or nmol/g·h)	half-life (h)	brain: Plasma ratio
**14**	5	IP	plasma	2.89 ± 0.57	0.25	1.56 ± 0.20	0.37	0.76
			brain	2.16 ± 0.21	0.25	1.19 ± 0.10	0.40	
**15**	5	IP	plasma	3.28 ± 1.26	0.25	2.65 ± 0.46	1.09	0.72
			brain	1.83 ± 0.68	0.25	1.91 ± 0.31	1.02	
**17**	5	IP	plasma	10.0 ± 1.27	0.25	15.1 ± 1.83	2.10	0.30
			brain	3.04 ± 0.45	0.25	4.57 ± 0.50	2.36	
**18**	10	IP	plasma	0.70 ± 0.09	0.50	1.36 ± 0.29	2.54	2.63
			brain	2.08 ± 0.20	0.50	3.57 ± 0.77	2.46	

## Conclusion

Evidence from prior studies indicates that D_4_R signaling may play important roles in cognition and attention, but major questions remain about how D_4_R signaling contributes to various neuropsychiatric disorders or the physiological consequences associated with the polymorphic nature of the human *DRD4* gene.^11,50^ Pharmacological targeting of D_4_Rs may be useful for treating cognitive deficits associated with neuropsychiatric disorders including schizophrenia and ADHD. D_4_R agonism has been explored as a strategy to reduce the adverse effects of opioid drugs like morphine. D_4_R antagonism may have potential to treat l-DOPA-induced dyskinesias and impulse-control disorders, including SUDs, eating disorders, and pathological gambling.^17,20^ The importance of targeting D_4_Rs in treating these complex pathologies, especially in regard to the extent of receptor activation or inhibition, remains unknown, partially due to a lack of suitable compounds for investigating these pathways.

In prior studies, we developed and characterized libraries of novel D_4_R ligands with high subtype selectivity and varying efficacies, from full antagonists to high-efficacy partial agonists.^32,51^ This study extends our previous work, employing a copper-catalyzed azide–alkyne cycloaddition click chemistry approach to improve the pharmacokinetic properties of previously reported compounds,^32^ making them more suitable for *in vivo* behavioral studies. This is a strategy we have previously employed successfully,^38^ simultaneously replacing a metabolically labile functional group while employing a new route to facile modular synthesis of novel libraries *via* click chemistry.

In this study, the bioisosteric replacement of amide linkers with a 1,2,3-triazole moiety resulted in modest improvements in D_4_R affinity when compared to their parent compounds, with minimal changes or modest improvements in D_2_-like subtype selectivity and CNS MPO scores (Table 1). Amide and triazole analogs had generally similar signaling profiles. 1,2,3-triazole analogs typically showed a small reduction in efficacy in β-arrestin BRET studies when compared to previously published values for the parent amides (Table 2). Similarly, at D_4_R, cAMP efficacy tended to be lower for 1,2,3-triazole analogs (Table 3) compared to amides. All ligands tend to show higher efficacy in cAMP assays than in β-arrestin association assays, consistent with our prior findings. This likely results from differences in experimental conditions, including signal amplification and the effects of receptor reserves across different assays. Binding affinities often differ from functional EC_50_/IC_50_ values for similar reasons. Although it is tempting to speculate, these studies are not comprehensive enough to evaluate possible biased signaling. This is, however, an interesting open question: little is known about whether D4R-mediated cAMP or β-arrestin signaling may differentially affect behavioral outcomes in animal models of cognition or substance use disorders.

Molecular modeling studies support the idea that the 1,2,3-triazole substitution minimally impacted ligand orientation in the binding site, with small improvements in binding energies consistent with improved D_4_R affinity in radioligand competition binding studies. 1,2,3-triazole analogs provided substantive gains in metabolic stability compared to matching amides, particularly in rat microsomal studies. Notably, the triazole substitution appears to have completely eliminated non-Phase I (NADPH−) metabolism of these compounds, which was a more substantial driver of metabolism in rat microsomes than in human microsomes. Prior work has demonstrated that 1,2,3-triazoles moieties can inhibit the enzymatic activity of hydrolase enzymes, including those with amidase activity,^52^ which could explain the increased stability of the triazole series compared to matching amide compounds in NADPH-free conditions.

Full characterization of triazole analogs **14**, **15**, **17**, and **18** show that each ligand has an adequate pharmacokinetic profile for behavioral testing. In particular, **17** (a full antagonist) and **18** (a low-efficacy partial agonist) had desirable results in plasma half-life and brain exposure measures. **18** demonstrated improved metabolic stability in both human and rat liver microsomes in comparison to its amide analog, with the longest *in vivo* plasma half-life and greatest brain penetration values in this study. Of note, behavioral studies using several of these compounds are presently underway in a variety of rodent models of different neuropsychiatric disorders.

Overall, this new 1,2,3-triazole analog library represents compounds with high D_4_R affinity, good selectivity over D_2_R and D_3_R, and a range of efficacy profiles. We are optimiztic that these analogs will be useful as improved *in vivo* research tools to explore the role of D_4_R signaling in a range of behavioral models of neuropsychiatric disorders.

## Experimental Methods

Reaction conditions and yields were not optimized. Anhydrous solvents were purchased from Sigma-Aldrich Corporation and were used without further purification. All other chemicals and reagents were purchased from Sigma-Aldrich Co. LLC, Aurora Fine Chemicals LLC, VWR Chemicals, Enamine, Acros Organics, and Alfa Aesar. All amine final products were converted into either oxalate or hydrochloride salt. Spectroscopic data and yields refer to the free base form of compounds. Flash chromatography was performed using silica gel (EMD Chemicals, Inc.; 230–400 mesh, 60 Å) by using a Teledyne ISCO CombiFlash RF system. ^1^H and ^13^C spectra were acquired using a JEOL ECZ-400S NMR spectrometer. All ^1^H and ^13^C NMR experiments are reported in δ units and were measured relative to the signals for CDCl_3_ (δ_H_ 7.26 ppm and δ_C_ 77.16 ppm), CD_2_Cl_2_ (δ_H_ 5.32 ppm and δ_C_ 53.84 ppm) or (CD_3_)_2_CO (δ_H_ 2.05 ppm and δ_C_ 29.84 and 206.26 ppm). Chemical shifts, multiplicities, and coupling constants (*J*) have been reported and calculated using MNova 64. Combustion elemental analysis was performed by Atlantic Microlab, Inc. (Norcross, GA) and the results agree within ± 0.4% of calculated values (Table S1). *c* Log *P* values were calculated using ChemDraw version 23.0. The CNS-MPO scores were calculated using ChemDraw, version 23.0 and ChemAxon Marvin version.^41,42^ Melting point determination was conducted using an SRS OptiMelt MPA100-Automated melting point apparatus and are uncorrected. Based on NMR and combustion elemental analysis data, all final compounds are ≥95% pure. Compounds **1–7** have been previously described in the peer-reviewed literature.^32^

### General Method A^38^

Propargyl *p*-toluenesulfonate (1 equiv) and the specific arylpiperidine or arylpiperazine (1 equiv) were dissolved in acetone. Potassium carbonate (2 equiv) and sodium iodide (5–10 mg) were added to the mixture. The reaction mixture was stirred at 60 °C overnight under N_2_ atmosphere. After the reaction was complete, the solvent was removed under reduced pressure. The product was purified by flash chromatography (50% EtOAc:Hexane) gradient to give the desired intermediates.

#### 4-Phenyl-1-(prop-2-yn-1-yl)­piperidine (**9**)

The compound was synthesized using propargyl *p*-toluenesulfonate (0.536 mL, 3.10 mmol), 4-phenylpiperidine (500 mg, 3.10 mmol), potassium carbonate (856.8 mg, 6.20 mmol) in acetone (12 mL) to yield dark orange solid (321 mg, 52%). ^1^H NMR (400 MHz, CDCl_3_) δ 7.36–7.11 (m, 5H), 3.35 (dd, *J* = 2.4, 0.8 Hz, 2H), 3.01 (dt, *J* = 12.3, 3.2 Hz, 1H), 2.58–2.44 (m, 1H), 2.41–2.22 (m, 4H), 1.91–1.70 (m, 4H).

#### 1-(Prop-2-yn-1-yl)-4-(pyridin-2-yl)­piperazine (**10**)

The compound was synthesized using propargyl *p*-toluenesulfonate (1.06 mL, 6.13 mmol), 1-(2-pyridyl)­piperazine (0.933 mL, 6.13 mmol), potassium carbonate (1.69 g, 12.25 mmol) in acetone (25 mL) to yield yellow solid (925.9 mg, 75%). ^1^H NMR (400 MHz, CDCl_3_) δ 8.17 (ddd, *J* = 5.0, 2.0, 0.9 Hz, 1H), 7.46 (ddd, *J* = 8.6, 7.1, 2.0 Hz, 1H), 6.67–6.58 (m, 2H), 3.61–3.52 (m, 4H), 3.35 (d, *J* = 2.5 Hz, 2H), 2.71–2.64 (m, 4H), 2.26 (t, *J* = 2.4 Hz, 1H).

#### 2-(4-(Prop-2-yn-1-yl)­piperazin-1-yl)­pyrimidine (**11**)

The compound was synthesized using propargyl *p*-toluenesulfonate (1.05 mL, 6.09 mmol), 2-(piperazin-1-yl)­pyrimidine (0.86 mL, 6.09 mmol), potassium carbonate (1.68 g, 12.18 mmol) in acetone (25 mL) to yield solid product (910 mg, 74%). ^1^H NMR (400 MHz, CD_2_Cl_2_) δ 8.28 (d, *J* = 4.7 Hz, 2H), 6.47 (t, *J* = 9.5, 4.7 Hz, 1H), 3.82 (dd, *J* = 10.2, 5.0 Hz, 4H), 3.33 (d, *J* = 2.5 Hz, 2H), 2.57 (dd, *J* = 10.3, 5.1 Hz, 4H), 2.29 (t, *J* = 2.5 Hz, 1H).

#### 1-(5-Chloropyridin-2-yl)-4-(prop-2-yn-1-yl)­piperazine (**12**)

The compound was synthesized using propargyl *p*-toluenesulfonate (0.88 mL, 5.06 mmol), 1-(5-chloropyridin-2-yl)­piperazine (1.0 g, 5.06 mmol), potassium carbonate (1.66 g, 10.12 mmol) in acetone (25 mL) to yield solid product (850 mg, 71%). ^1^H NMR (400 MHz, (CD_3_)_2_CO) δ 8.07 (dd, *J* = 2.6, 0.7 Hz, 1H), 7.52 (ddd, *J* = 9.0, 2.6, 0.7 Hz, 1H), 6.83 (d, *J* = 9.0 Hz, 1H), 3.56 (dd, *J* = 10.2, 5.1 Hz, 4H), 3.36 (d, *J* = 2.5 Hz, 2H), 2.73 (t, *J* = 2.4 Hz, 1H), 2.60 (dd, *J* = 10.2, 5.1 Hz, 4H).

#### 1-(Naphthalen-1-yl)-4-(prop-2-yn-1-yl)­piperazine (**13**)

The compound was synthesized using propargyl *p*-toluenesulfonate (0.408 mL, 2.36 mmol), 1-(naphthalen-1-yl)-piperazine (500 mg, 2.36 mmol), potassium carbonate (650.92 mg, 4.71 mmol) in acetone (12 mL) to yield light yellow solid (430 mg, 73%). ^1^H NMR (400 MHz, CDCl_3_) δ 8.23–8.16 (m, 1H), 7.84–7.77 (m, 1H), 7.54 (d, *J* = 8.1 Hz, 1H), 7.50–7.42 (m, 2H), 7.39 (dd, *J* = 8.2, 7.4 Hz, 1H), 7.09 (dd, *J* = 7.4, 1.1 Hz, 1H), 3.43 (d, *J* = 2.5 Hz, 2H), 3.18 (s, 4H), 2.87 (s, 4H), 2.32 (t, *J* = 2.4 Hz, 1H).

### General Method B

The specific 1-azidobenzene (1 equiv) and the intermediate (1.0 equiv) were dissolved in a water/tert-butanol mixture. Sodium ascorbate (0.1 equiv) and copper­(II) sulfate pentahydrate (0.01 equiv) were individually dissolved in H_2_O and added to the solution. The heterogeneous mixture was stirred at room temperature overnight under N_2_ atmosphere. After the reaction was complete, the solvent was removed under reduced pressure. The product was subjected to flash column chromatography to provide the desired compounds. All final products were converted into oxalate salts.

#### 4-Phenyl-1-((1-(*m*-tolyl)-1*H*-1,2,3-triazol-4-yl)­methyl)­piperidine (**14**)

The compound was synthesized using 1-azido-3-methylbenzene (222.2 mg, 1.51 mmol), 4-phenyl-1-(prop-2-yn-1-yl)­piperidine (**9**) (1.51 mmol, 200 mg), sodium ascorbate (30 mg, 0.15 mmol), copper­(II) sulfate pentahydrate (3.7 mg, 0.015 mmol) in a mixture of tert-butanol (0.3 g) and H_2_O (8 mL). The product was purified by flash column chromatography (60% EtOAc/Hexane) to yield an orange solid (306.2 mg, 61%). ^1^H NMR (400 MHz, CD_2_Cl_2_) δ 7.97 (s, 1H), 7.64–7.60 (m, 1H), 7.55 (dd, *J* = 8.0, 2.2 Hz, 1H), 7.42 (t, *J* = 7.8 Hz, 1H), 7.32–7.22 (m, 5H), 7.18 (ddt, *J* = 7.3, 5.7, 1.3 Hz, 1H), 3.75 (s, 2H), 3.09 (d, *J* = 11.8 Hz, 2H), 2.53 (tt, *J* = 11.5, 4.5 Hz, 1H), 2.46 (s, 3H), 2.21 (td, *J* = 11.4, 3.2 Hz, 2H), 1.81 (qd, *J* = 12.6, 3.6 Hz, 4H). ^13^C NMR (101 MHz, CD_2_Cl_2_) δ 146.94, 146.03, 140.46, 137.54, 129.82, 129.58 (2C), 128.70 (2C), 127.20, 126.39, 121.29, 121.21, 117.69, 56.03, 54.46, 53.96, 42.79, 33.89 (2C), 21.52. The oxalate salt was precipitated from 2-propanol. Mp: 216.5–217.2 °C. Anal. (C_21_H_24_N_4_•C_2_H_2_O_4_) C, H, N.

#### 1-(Pyridin-2-yl)-4-((1-(*m*-tolyl)-1*H*-1,2,3-triazol-4-yl)­methyl)­piperazine (**15**)

The compound was synthesized using 1-azido-3-methylbenzene (500 mg, 3.755 mmol), 1-(prop-2-yn-1-yl)-4-(pyridin-2-yl)­piperazine (**10**) (750 mg, 3.755 mmol), sodium ascorbate (75 mg, 0.3755 mmol), copper­(II) sulfate pentahydrate (10 mg, 0.03755 mmol) in a mixture of *tert*-butanol (0.5 g) and H_2_O (12 mL). The product was purified by flash column chromatography (95% EtOAc/Hexane) to yield a clay-colored crude product (886.5 mg, 69%). ^1^H NMR (400 MHz, CD_2_Cl_2_) δ 8.13 (t, *J* = 2.3 Hz, 1H), 7.98 (d, *J* = 8.1 Hz, 1H), 7.59 (d, *J* = 7.0 Hz, 1H), 7.55–7.36 (m, 3H), 7.26 (t, *J* = 7.6 Hz, 1H), 6.64 (t, *J* = 8.8 Hz, 1H), 6.58 (ddd, *J* = 8.5, 5.5, 3.1 Hz, 1H), 3.77 (d, *J* = 7.7 Hz, 2H), 3.53 (dq, *J* = 8.5, 4.6 Hz, 4H), 2.68–2.61 (m, 4H), 2.44 (d, *J* = 7.2 Hz, 3H). ^13^C NMR (101 MHz, CD_2_Cl_2_) δ 159.90, 148.21, 148.19, 145.34, 140.51, 137.66, 129.85, 129.67, 121.44, 121.35, 117.76, 113.47, 107.25, 53.65, 53.12 (2C), 45.42 (2C), 21.53. The oxalate salt was precipitated from 2-propanol. Mp: 227.3–228.1 °C. Anal. (C_19_H_22_N_6_·C_2_H_2_O_4_) C, H, N.

#### 2-(4-((1-(*m*-Tolyl)-1*H*-1,2,3-triazol-4-yl)­methyl)­piperazin-1-yl)­pyrimidine (**16**)

The compound was synthesized using 1-azido-3-methylbenzene (463 mg, 3.46 mmol), 2-(4-(prop-2-yn-1-yl)­piperazin-1-yl)­pyrimidine (**11**) (700 mg, 3.46 mmol), sodium ascorbate (68.5 mg, 0.346 mmol), copper­(II) sulfate pentahydrate (8.64 mg, 0.0346 mmol) in a mixture of tert-butanol (0.5 g) and H_2_O (10 mL). The product was purified by flash column chromatography (90% EtOAc/Hexane) to yield a red solid (870.4 mg, 75%). ^1^H NMR (400 MHz, CD_2_Cl_2_) δ 8.27 (t, *J* = 3.7 Hz, 2H), 7.97 (d, *J* = 2.7 Hz, 1H), 7.58 (s, 1H), 7.52 (d, *J* = 8.3 Hz, 1H), 7.40 (td, *J* = 7.9, 2.5 Hz, 1H), 7.25 (d, *J* = 7.6 Hz, 1H), 6.46 (q, *J* = 4.0 Hz, 1H), 3.80 (p, *J* = 4.8 Hz, 4H), 3.75 (d, *J* = 2.5 Hz, 2H), 2.57 (t, *J* = 4.8 Hz, 4H), 2.44 (s, 3H). ^13^C NMR (101 MHz, CD_2_Cl_2_) δ 162.10, 157.98 (2C), 145.38, 140.48, 140.47, 137.44, 129.82, 129.63, 121.37, 121.29, 117.69, 110.14, 53.57 (2C), 43.91 (2C), 21.51. The oxalate salt was precipitated from 2-propanol. Mp: 224 −224.5 °C. Anal. (C_18_H_21_N_7_•C_2_H_2_O_4_) C, H, N.

#### 1-(5-Chloropyridin-2-yl)-4-((1-(*m*-tolyl)-1*H*-1,2,3-triazol-4-yl)­methyl)­piperazine (**17**)

The compound was synthesized using 1-azido-3-methylbenzene (398 mg, 2.97 mmol), 1-(5-chloropyridin-2-yl)-4-(prop-2-yn-1-yl)­piperazine (**12**) (700 mg, 2.97 mmol), sodium ascorbate (55.27 mg, 0.297 mmol), copper­(II) sulfate pentahydrate (7.49 mg, 0.0297 mmol) in a mixture of tert-butanol (0.5 g) and H_2_O (10 mL). The product was purified by flash column chromatography (80% EtOAc/Hexane) to yield a white solid (624.5 mg, 57%). ^1^H NMR (400 MHz, CD_2_Cl_2_) δ 8.07 (d, *J* = 2.6 Hz, 1H), 7.96 (s, 1H), 7.59 (s, 1H), 7.52 (d, *J* = 8.3 Hz, 1H), 7.46–7.37 (m, 2H), 7.27 (d, *J* = 7.6 Hz, 1H), 6.60 (d, *J* = 9.1 Hz, 1H), 3.77 (s, 2H), 3.52 (t, *J* = 5.1 Hz, 4H), 2.62 (t, *J* = 5.0 Hz, 4H), 2.45 (s, 3H). ^13^C NMR (101 MHz, CD_2_Cl_2_) δ 158.16, 146.36, 145.27, 140.46, 137.40, 137.26, 129.81, 129.63, 121.37, 121.26, 120.10, 117.66, 108.03, 53.54, 52.89 (2C), 45.46 (2C), 21.50. The oxalate salt was precipitated from 2-propanol. Mp: 222.8–223.1 °C. Anal. (C_19_H_21_ClN_6_·C_2_H_2_O_4_) C, H, N.

#### 1-(Naphthalen-1-yl)-4-((1-(*m*-tolyl)-1*H*-1,2,3-triazol-4-yl)­methyl)­piperazine (**18**)

The compound was synthesized using 1-azido-3-methylbenzene (212.98 mg, 1.59 mmol), 1-(naphthalen-1-yl)-4-(prop-2-yn-1-yl)­piperazine (**13**) (1.59 mmol, 400 mg), sodium ascorbate (31.5 mg, 0.159 mmol), copper­(II) sulfate pentahydrate (4.0 mg, 0.0159 mmol) in a mixture of tert-butanol (0.5 g) and H_2_O (8 mL). The product was purified by flash column chromatography (70% EtOAc/Hexane) to yield an orange/brown solid (323.2 mg, 53%). ^1^H NMR (400 MHz, CD_2_Cl_2_) δ 8.23–8.18 (m, 1H), 8.02 (d, *J* = 1.8 Hz, 1H), 7.85–7.80 (m, 1H), 7.63 (d, *J* = 2.2 Hz, 1H), 7.55 (dd, *J* = 8.4, 3.8 Hz, 2H), 7.52–7.45 (m, 2H), 7.45–7.37 (m, 2H), 7.27 (d, *J* = 7.6 Hz, 1H), 7.11 (d, *J* = 7.4 Hz, 1H), 3.88 (d, *J* = 1.7 Hz, 2H), 3.16 (s, 4H), 2.87 (s, 4H), 2.46 (s, 3H). ^13^C NMR (101 MHz, CD_2_Cl_2_) δ 150.04, 145.41, 140.51, 137.53, 135.13, 129.85, 129.66, 129.19, 128.65, 126.23, 126.14, 125.63, 123.98, 123.66, 121.46, 121.35, 117.75, 114.97, 53.74, 53.62 (2C), 53.30 (2C), 21.54. The oxalate salt was precipitated from 2-propanol. Mp: 184.1–184.9 °C. (C_24_H_25_N_5_·C_2_H_2_O_4_) C, H, N.

#### 1-((1-(3-Ethylphenyl)-1*H*-1,2,3-triazol-4-yl)­methyl)-4-(pyridin-2-yl)­piperazine (**19**)

The compound was synthesized using 1-azido-3-ethylbenzene (441.54 mg, 3 mmol), 1-(prop-2-yn-1-yl)-4-(pyridin-2-yl)­piperazine (**10**) (603.81 mg, 3 mmol), sodium ascorbate (59.43 mg, 0.3 mmol), copper­(II) sulfate pentahydrate (7.5 mg, 0.03 mmol) in a mixture of tert-butanol (0.5 g) and H_2_O (10 mL). The product was purified by flash column chromatography (80% EtOAc/Hexane) to yield a transparent solid (512.2 mg, 49%). ^1^H NMR (400 MHz, CD_2_Cl_2_) δ 8.14–8.10 (m, 1H), 7.98 (s, 1H), 7.60 (s, 1H), 7.53 (d, *J* = 8.1 Hz, 1H), 7.48–7.40 (m, 2H), 7.28 (d, *J* = 7.6 Hz, 1H), 6.63 (d, *J* = 8.6 Hz, 1H), 6.58 (dd, *J* = 7.1, 4.9 Hz, 1H), 3.76 (s, 2H), 3.52 (t, *J* = 5.1 Hz, 4H), 2.74 (q, *J* = 7.6 Hz, 2H), 2.63 (t, *J* = 5.1 Hz, 4H), 1.27 (t, *J* = 7.6 Hz, 3H). ^13^C NMR (101 MHz, CD_2_Cl_2_) δ 159.89, 148.18, 146.82, 145.41, 137.64, 137.56, 129.92, 128.52, 121.42, 120.25, 117.98, 113.43, 107.22, 53.65, 53.11 (2C), 45.41 (2C), 29.10, 15.60. The oxalate salt was precipitated from 2-propanol. Mp: 212.1–212.7 °C. (C_20_H_24_N_6_•C_2_H_2_O_4_) C, H, N.

### Radioligand Binding Assays

Binding at dopamine D_2_-like receptors was determined similarly to previously described methods,^53^ and identical to the methods previously used in Keck and Free et al.^32^ Membranes were prepared from HEK293 cells stably expressing human D_2L_R, D_3_R, or D_4_R grown in a 50:50 mix of DMEM and Ham’s F12 culture media, supplemented with 20 mM HEPES, 2 mM l-glutamine, 0.1 mM nonessential amino acids, 1× antibiotic/antimycotic, 10% heat-inactivated fetal bovine serum, and 200 μg/mL hygromycin (Life Technologies, Grand Island, NY) and kept in an incubator at 37 °C and 5% CO_2_. Upon reaching 80–90% confluence, cells were harvested using premixed Earle’s Balanced Salt Solution (EBSS) with 5 mM EDTA (Life Technologies) and centrifuged at 3,000 rpm for 10 min at 21 °C. The supernatant was removed, and the pellet was resuspended in 10 mL hypotonic lysis buffer (5 mM MgCl_2_·6H_2_O, 5 mM Tris, pH 7.4 at 4 °C) and centrifuged at 14,500 rpm (∼25,000*g*) for 30 min at 4 °C. The pellet was then resuspended in fresh EBSS binding buffer made from 8.7 g/L Earle’s Balanced Salts without phenol red (US Biological, Salem, MA), 2.2 g/L sodium bicarbonate, pH to 7.4. A Bradford protein assay (Bio-Rad, Hercules, CA) was used to determine the protein concentration and membranes were diluted to 500 μg/mL and stored in a −80 °C freezer for later use.

Radioligand competition binding experiments were conducted using freshly dissolved drugs on each test day. Each test compound was diluted into 10 half-log serial dilutions using 30% DMSO vehicle, ranging from 100 μM to 0.3 nM final concentrations, adjusted depending on compound solubility and to optimize binding curve calculations. Previously frozen membranes were thawed and diluted in fresh EBSS binding buffer to 200 μg/mL (for hD_2L_R or hD_3_R) or 400 μg/mL (for hD_4_R) for binding. Radioligand competition reactions were conducted in 96-well plates containing 300 μL fresh EBSS binding buffer, 50 μL of diluted test compound, 100 μL of diluted membranes (20 μg/well total protein for hD_2L_R and hD_3_R, or 40 μg/well total protein for hD_4_R), and 50 μL of [^3^H]*N*-methylspiperone radioligand diluted in binding buffer (0.4 nM final concentration; PerkinElmer). Nonspecific binding was determined using 10 μM (+)-butaclamol (Sigma-Aldrich, St. Louis, MO) and total binding was determined with 30% DMSO vehicle. All compound dilutions were tested in triplicate and the reaction incubated for 1 h at RT. The reaction was terminated by filtration through PerkinElmer Uni-Filter-96 GF/B plates, presoaked for 1 h in 0.5% polyethylenimine, using a Brandel 96-Well Plates Harvester Manifold (Brandel Instruments, Gaithersburg, MD). The filters were washed (3 × 1 mL/well) with ice-cold binding buffer. After drying overnight at RT, PerkinElmer MicroScint 20 Scintillation Cocktail (45 μL) was added to each well and filters were counted using a PerkinElmer MicroBeta2 scintillation counter. IC_50_ values for each compound at each receptor were determined from dose–response curves and *K_i_* values were calculated using the Cheng-Prusoff equation.^54^ When a complete inhibition could not be achieved at the highest tested concentrations, *K_i_* values have been extrapolated by constraining the bottom of the dose–response curves (=0% residual specific binding) in the nonlinear regression analysis. These analyses were performed using GraphPad Prism versions 6.00–8.00 (GraphPad Software, San Diego, CA). All results were rounded to three significant figures. *K_i_* values were determined from at least 3 independent experiments and are reported as means ± SEM.

### Functional Assays

#### β-Arrestin Recruitment Assay

Assays were conducted with minor modifications as previously published by our laboratory,^2,19–23^ and identical to the methods previously used,^32^ using the DiscoverX PathHunter technology (Eurofins DiscoverX, Fremont, CA). Briefly, CHO-K1 cells stably expressing the human D_2_R long isoform, D_3_R, or D_4_R (Eurofins DiscoverX) were maintained in Ham’s F12 media supplemented with 10% fetal bovine serum, 100 U/mL penicillin, 100 μg/mL streptomycin, 800 μg/mL G418 and 300 μg/mL hygromycin at 37 °C, 5% CO_2_, and 90% humidity. The cells were seeded in 7.5 μL media at a density of 2,625 cells/well in 384-well black, clear-bottom plates. The following day, the compounds were diluted in PBS with 0.2 mM sodium metabisulfite. The cells were treated with 16 concentrations of a compound in triplicate and incubated at 37 °C for 90 min. Tropix Gal-Screen Substrate (Applied Biosystems, MA) was diluted in Gal-Screen buffer A (Applied Biosystems) 1:25 and added to cells according to the manufacturer’s recommendations followed by a 30–45 min incubation at room temperature in the dark. Luminescence was measured on a Hamamatsu FDSS μCell reader. Data were collected in triplicate and transferred to GraphPad Prism 9 where it was fit with nonlinear regression curve fit equations. The data were normalized to the percent maximum dopamine response (agonist mode) or the EC_80_ of dopamine (antagonist mode). The Hill coefficients of the concentration–response curves did not significantly differ from unity with the data fitting to a single site model. Data in Table 2 are from at least three independent replicates. The data from each experiment was fit as described above with the *E*_max_, Ant.%, EC_50_, and IC_50_ values extracted from the nonlinear regression. The *E*_max_, Ant. %, EC_50_ and IC_50_ values were meaned together using descriptive statistics in Prism and reported as mean ± SEM. Fold selectivity for the D_4_R over the D_2_R and D_3_R were also calculated and presented in Table 2.

#### cAMP Inhibition Assay

D_4_R-mediated inhibition of forskolin-stimulated cAMP production was assayed using the PerkinElmer LANCE Ultra cAMP assay kit (PerkinElmer, Inc., Waltham, MA). CHO-K1 cells stably expressing the human D4R were maintained in Ham’s F12 supplemented with 10% fetal bovine serum, 100 U/mL penicillin, 100 μg/mL streptomycin, 800 μg/mL G418, and 300 μg/mL hygromycin at 37 °C, 5% CO_2_, and 90% humidity. Cells were seeded in Hank’s balanced salt solution (with CaCl_2_ and MgCl_2_) with 5 mM HEPES buffer and 0.2 μM sodium metabisulfite at a density of 5000 cells/well in 384-well white plates. Compounds and forskolin were made in the same buffer. Immediately after plating, cells were treated with 2.5 μL of compound (at various concentrations) and 2.5 μL of forskolin and incubated at room temperature for 30 min. The final concentration of forskolin was 10 μM. When running assay in antagonist mode, the EC_80_ of dopamine (10 nM final concentration) was added with the compound dilution buffer. Eu-cAMP tracer and ULight-anti-cAMP solutions were added as directed by the manufacturer and cells were incubated for 2 h in the dark at room temperature, after which a time-resolved fluorescence resonance energy transfer (TR-FRET) signal was measured using a BMG Labtech PHERAstar Fs (BMG Labtech USA, Cary, NC). Values were normalized to a percentage of the control TR-FRET signal seen with a maximum concentration of dopamine for agonist mode assays and the EC_80_ of dopamine for antagonist mode assays. The Hill coefficients of the concentration–response curves did not significantly differ from unity with the data fitting to a single site model. Data in Table 3 are from at least three independent replicates. The data from each experiment was fit, as described above, with the *E*_max_, Ant. %, EC_50_, and IC_50_ values extracted from the nonlinear regression. These values were then averaged together across experiments using descriptive statistics in Prism and reported as means ± SEM.

### Molecular Modeling and Docking

#### Model and Ligand Preparation

To initiate the molecular dynamics (MD), the initial crystal structure was obtained from the RCSB PDB Web site. The crystal structure obtained was PDB ID 5WIU^43^ which has a resolution of 2.6 Å. The T4-lysozyme that is used to take the place of the intracellular loop 3 was deleted and replaced with *N*-methyl and acetyl caps on the termini of this deleted region. All molecules in the PDB were deleted except for the receptor. The protonation states of ionizable residues were assigned by the H++ server^55^ with pH 7.4.

l-Dopamine was used as the ligand for the initial MD simulation. The model for l-dopamine was generated using the 2D sketcher function of Schrodinger’s Maestro, the Ligprep protocol was used to generate conformations of this ligand and protonation states using a pH of 7.4 ± 2.0.^56^ Twenty-five generated molecules were requested and only one conformation generated the positively charged amine, which was kept.

The model was placed into Schrodinger’s Maestro for visualization, followed by their protein preparation protocol, and finally receptor grid generation as part of their docking protocol. The receptor grid was formed using residue D115^3.32^ as the center. Dopamine was docked into the D_4_R. The highest scoring pose was kept which resembles the binding mode of dopamine in literature.

The receptor was given to Packmol-memgen^57^ to create a lipid membrane for the simulation. Lipids were generated in a 9:1 ratio of POPC:CHL1, respectively. Additional ions to mimic a salt concentration of 150 mm NaCl were added by packmol-memgen. Antechamber^58^ was used to assign a +1 charge to l-dopamine. The tleap^59^ module was used to prepare the system. Tleap used the Amber FF19SB force field^60^ for the protein, OPC water model,^61^ gaff2 ^62^ for the ligand, and lipid21 ^63^ for the membrane. Parmed^64^ was utilized to activate hydrogen mass repartitioning (HMR) which allows for a 4 fs (fs) time step.

#### Molecular Dynamics Simulations

MD simulations were performed using the AMBER^59^ suite. The model underwent five minimization steps. First, the model underwent 5000 cycles of steep descent, followed by 5000 cycles of conjugate gradient with a restraint weight of 25 kcal·mol^–1^·Å^–2^ on the membrane and protein. Next, the model underwent 5000 cycles of steep descent, followed by 5000 cycles of conjugate gradient with a restraint weight of 5 kcal·mol^–1^·Å^–2^ on the membrane and protein. In the third minimization step, the model underwent 5000 cycles of steep descent, followed by 5000 cycles of conjugate gradient with a restraint weight of 5 kcal·mol^–1^·Å^–2^ on the protein. In the fourth minimization step, the model underwent 5000 cycles of steep descent, followed by 5000 cycles of conjugate gradient with a restraint weight of 1 kcal·mol^–1^·Å^–2^ on the protein. In the fifth minimization step, the model underwent 5000 cycles of steep descent, followed by 10,000 cycles of conjugate gradient with no restraints.

The SHAKE algorithm was applied to all bonds connected to hydrogen atoms with a time step of 4 fs. The system was heated from 0 to 100 K in 5 ps (ps) with restraints of 5 kcal·mol^–1^·Å^–2^ on the membrane and protein. The Langevin thermostat^65^ was used with a collision frequency value of 2.0 ps and cutoff of 10.0 Å. The system then underwent additional heating to 310 K over 100 ps with restraints still held. The Berendsen barostat^66^ was used during the equilibration process which occurred in three steps. First, restraints of 5 kcal·mol^–1^·Å^–2^ were placed on the ligand and the protein backbone for 2 ns (ns). Next, restraints of 5 kcal·mol^–1^·Å^–2^ were placed on the ligand and the α carbons of the protein for 2 ns. Lastly, all atoms were allowed to move freely for 100 ns prior to the production run. The Monte Carlo barostat^67^ was then used for the production run with a target pressure of 1 atm. The production ran for 2.5 μs (μs).

#### Docking Studies

After 2.5 μs of MD simulations, frames of the trajectory were manually visualized to obtain a frame with the extended binding pocket (EBP) visible. A frame from the first 100 ns was used for docking purposes. The model was placed into Schrodinger’s Maestro for visualization, followed by their protein preparation protocol, and finally receptor grid generation as part of their docking protocol. The receptor grid was formed using residue D115^3.32^ as the center. The amide-based and triazole-based compounds were drawn using the 2D sketcher functionality and converted to 3D structures. The compounds underwent Maestro’s LigPrep protocol using a pH of 7.4 ± 2.0 ^56^ and was asked to generate 20 conformers. Four conformations of each compound were generated with only one containing the positively charged amine so one of the four was kept while the others were discarded. This conformation was used for the Schrodinger Glide SP Protocol^68^ and docked into the D_4_R. With 50 poses requested, 15 were generated.

#### DeepAtom Binding Energy Analysis

The compounds coinciding with the highest reported docking score were converted to pdbqt files using Obabel.^69^ After this, DeepAtom^44^ was used to predict the binding energies of the compounds.

### Rat and Human Microsomal Stability Assays

Phase I metabolic stability assays were conducted using rat and human liver microsomes as previously described^45,70^ with minor modifications. In brief, the reactions were carried out with 100 mM potassium phosphate buffer, pH 7.4, in the presence of NADPH regenerating system (1.3 mM NADPH, 3.3 mM glucose 6-phosphate, 3.3 mM MgCl_2_, 0.4 U/mL glucose-6-phosphate dehydrogenase, 50 μM sodium citrate). Negative controls without cofactors were assessed to determine the non-CYP-mediated metabolism. Compound disappearance was monitored over time using a liquid chromatography and tandem mass spectrometry (LC/MS) method. All reactions were performed in triplicate.

Chromatographic analysis was performed on a Dionex ultra high-performance LC system coupled with Q Exactive Focus orbitrap mass spectrometer (Thermo Fisher Scientific Inc., Waltham MA). Separation was achieved using Agilent Eclipse Plus column (100 × 2.1 mm^2^ i.d.; maintained at 35 °C) packed with a 1.8 μm C18 stationary phase. The mobile phase used was composed of 0.1% Formic Acid in Acetonitrile and 0.1% Formic Acid in water with gradient elution, starting with 2.5% organic phase (from 0 to 2 min) linearly increasing to 99% (from 2 to 5.5 min), and re-equilibrating to 2.5% by 6.5 min. The total run time for each analyte was 6.5 min. Pumps were operated at a flow rate of 0.3 mL/min. The mass spectrometer controlled by Xcalibur software 4.0.27.13 (Thermo Scientific) was operated with a HESI ion source in positive ionization mode. Compounds were identified in the full-scan mode (from *m*/*z* 50 to 750) by comparing *t* = 0 samples with *t* = 30 min and *t* = 60 min samples.

### Pharmacokinetics Study in Rats

All animal experiments were performed following the protocols evaluated and approved by the Animal Care and Use Committee at Johns Hopkins University (Ethics Approval Number: RA24M403). Pharmacokinetic studies in Sprague–Dawley (SD) rats were conducted according to protocols approved. SD rats obtained from Harlan were maintained on a 12 h light–dark cycle with *ad libitum* access to food and water. Test compound was administered *via* i.p. injection at a dose of 10 mg/kg (100% saline vehicle, 10 mL/kg volume). The rats were sacrificed at specified time points (0.25, 0.5 h, 1, 2, 4, and 6 h) post drug administration. For the collection of plasma and brain tissue, animals were euthanized with CO_2_, and blood samples were collected in heparinized microtubes by cardiac puncture. Brains were dissected and immediately flash-frozen (−80 °C). Blood samples were spun at 2000*g* for 15 min, and plasma was removed and stored at −80 °C until analysis (as described below).

#### Bioanalysis

Quantitation of triazole analogs **14**, **15**, **17**, and **18** was performed using liquid chromatography with tandem mass spectrometry (LC/MS-MS) methods. Briefly, calibration standards were prepared using respective tissue (naïve plasma and brain) with additions of the test compounds. For quantifying the test compounds in the pharmacokinetic samples, plasma samples (20 μL) were processed using a single liquid extraction method by addition of 100 μL of acetonitrile containing internal standard (losartan: 0.5 μM), followed by vortex-mixing for 30 s and then centrifugation at 10,000*g* for 10 min at 4 °C. Brain tissues were diluted 1:5 w/v with acetonitrile containing losartan (0.5 μm) and homogenized, followed by vortex-mixing and centrifugation at 10,000*g* for 10 min at 4 °C. A 50 μL aliquot of the supernatant was diluted with 50 μL of water and transferred to 250 μL polypropylene autosampler vials sealed with Teflon caps. Two μL of the sample was injected into the LC/MS/MS system for analysis. Chromatographic analysis was performed using an Accela ultra high-performance system consisting of an analytical pump and an autosampler coupled with a TSQ Vantage mass spectrometer. Separation of the analyte was achieved at ambient temperature using an Agilent Eclipse Plus column (100 × 2.1 mm^2^ i.d.) packed with a 1.8 μm C18 stationary phase. The mobile phase consisted of 0.1% formic acid in acetonitrile and 0.1% formic acid in water with gradient elution, starting with 10% organic phase (from 0 to 1 min) linearly increasing to 95% (from 1 to 2 min), and re-equilibrating to 10% by 3 min. The total run time for each analyte was 3.5 min. Pumps were operated at a flow rate of 0.4 mL/min. The [M + H]^+^ ion transition of test compound **18** (*m*/*z* 384.2 □ 144.1, 182.1, 225.1) and losartan (IS) (*m*/*z* 423.2 □ 207.1, 377.2) were used. Plasma concentrations (nmol/mL) as well as brain tissue concentrations (nmol/g) were determined and plots of mean plasma concentration *versus* time were constructed. Noncompartmental analysis modules in Phoenix WinNonlin version 7.0 (Certara USA, Inc., Princeton, NJ) were used to quantify exposures (AUC_0–*t*_) and half-life (*t*_1/2_).

## Supplementary Material



## Notes

Elemental analyses are provided for all final compounds, including HPLC and MS traces of compounds **2–7** and **14–19**, ^1^H–^13^C NMR spectra of **14–19** (PDF)

## Abbreviations Used

CDCl_3_: deuterated chloroform
CD_2_Cl_2_: deuterated dichloromethane
(CD_3_)_2_CO: deuterated acetone
EtOAc: ethyl acetate
DA: dopamine
D_2_R: dopamine D_2_ receptor
D_3_R: dopamine D_3_ receptor
D_4_R: dopamine D_4_ receptor
NMR: nuclear magnetic resonance
RT: room temperature
