# Supplementary material for: Bioisosteric Replacement of Amides with 1,2,3-Triazoles Improves Dopamine D4 Receptor Ligand Pharmacokinetics
Source: ACS Pharmacol Transl Sci. Author manuscript; Available in PMC 2026 Mar 6. (PMC12910498; doi:10.1021/acsptsci.5c00646)
Supplement: Supplemental Material [file NIHMS2146938-supplement-Supplemental_Material.pdf]

Supporting Information

Bioisosteric Replacement of Amides with 1,2,3-Triazoles Improves Dopamine D4 Receptor Ligand Pharmacokinetics

Mohammad Alkhatib,[+,#] Franziska M. Jakobs,[†,#] John N. Hanson,[≠] Ashley N. Nilson,[≠] Amy E. Moritz,[≠] Tian Li,[†] Afua B. Faibille,[†] Lindsay A. Bourn,[†] Peter A. Ramdhan,[°] Joseph Ricchezza IV,[+] Shannon Jordan,[+] Diandra Panasis,[+] Norman Nguyen,[+] Nitish Kasarla,[+] Bryant Wang,[+] Sergio Sola Garcia,[+] Julianna Saez,[+] James Paule,[‡] Chae Bin Lee,[‡] Rana Rais,[‡] Barbara S. Slusher,[‡] David R. Sibley,[≠] Chenglong Li,[°] Thomas M. Keck,[+,] Comfort A. Boateng,[†,*]*

[†]Department of Basic Pharmaceutical Sciences, Fred Wilson School of Pharmacy, High Point University, One University Parkway, High Point, North Carolina 27268, United States

[+]Department of Chemistry & Biochemistry, Department of Biological & Biomedical Sciences, College of Science and Mathematics, Rowan University, 201 Mullica Hill Road, Glassboro, New Jersey 08028, United States

[‡]Department of Neurology, Johns Hopkins Drug Discovery, The Johns Hopkins University School of Medicine, 855 N. Wolfe Street, Baltimore, Maryland 21205, United States

[≠]Molecular Neuropharmacology Section, National Institute of Neurological Disorders and Stroke-Intramural Research Program, National Institutes of Health, Bethesda, Maryland 20892, United States

° Department of Medicinal Chemistry, University of Florida College of Pharmacy, 1345 Center Drive, Gainesville, Florida 32610, United States

[#] Equally Contributing Authors, * Corresponding Authors

Corresponding Authors

*Phone: (336) 841-9718. Fax: (336) 888-6354. E-mail: cboateng@highpoint.edu

*Phone: (856) 256-5422. E-mail: keckt@rowan.edu

Supporting information Contents:

Content	Page
**Table S1. Microanalysis data of the final compounds 14-19**	**S-4**
**Figure S1. HPLC and MS traces of compounds 2-4**	**S-5**
**Figure S2. HPLC and MS traces of compounds 5-6**	**S-6**
**Figure S3. HPLC and MS traces of compound 7**	**S-7**
**Figure S4. HPLC and MS traces of compounds 14-16**	**S-8**
**Figure S5. HPLC and MS traces of compounds 17-19**	**S-9**
**Figure S6. ^{1}H and ^{13}C NMR Spectra Data of compound 14**	**S-10**
**Figure S7. ^{1}H and ^{13}C NMR Spectra Data of compound 15**	**S-11**
**Figure S8. ^{1}H and ^{13}C NMR Spectra Data of compound 16**	**S-12**
**Figure S9. ^{1}H and ^{13}C NMR Spectra Data of compound 17**	**S-13**
**Figure S10. ^{1}H and ^{13}C NMR Spectra Data of compound 18**	**S-14**
**Figure S11. ^{1}H and ^{13}C NMR Spectra Data of compound 19**	**S-15**
**Table S2. Compounds (14, 15, 17, 18) effects on D_1-like dopamine receptors**	**S-16**
**Figure S12. Image of the D4R surface and aerial view of the binding pocket docked with matching representatives of triazole-based (17; top inset) and amide-based (2; bottom inset) analog sets.**	**S-17**
**Figure S13. Binding poses for compound 14 in the "opposite pose" (A) and the "consistent pose" (B).**	**S-18**
**Figure S14. Binding poses for compound 5 in the "opposite pose" (A) and the "consistent pose" (B).**	**S-19**

Table S1. Microanalysis data of the final compounds 14-19.

Compound	C	H	N	C	H	N
	Calculated			Found		
**14**	65.39	6.20	13.26	65.13	6.17	13.21
**15**	59.42	5.70	19.80	59.48	5.81	19.71
**16**	56.46	5.45	23.05	56.34	5.51	22.85
**17**	54.96	5.05	18.31	54.73	5.05	18.10
**18**	65.67	6.15	14.73	65.52	5.98	14.82
**19**	60.26	5.98	19.17	60.14	5.99	19.01

Figure S1. HPLC traces and MS traces of compounds 2-4. Note the metabolite identifications for **3**, showing dealkylated metabolic products with cleavage occurring at the amide linker.

Compound 2 (CAB02-140)

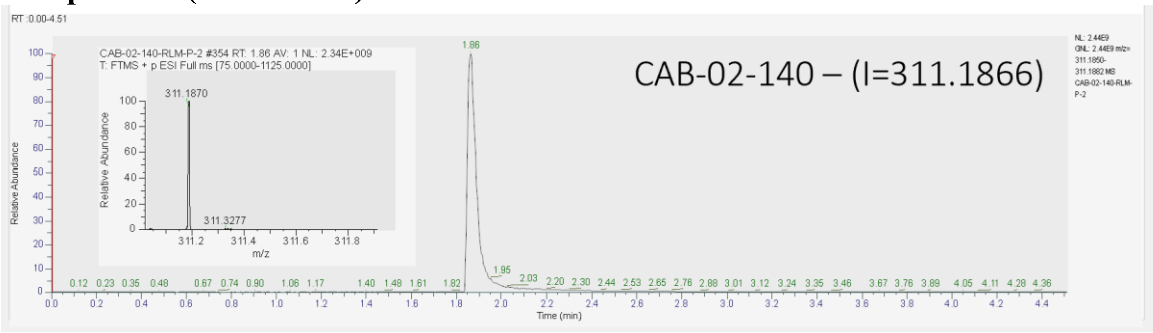

Compound 3 (CAB02-017)

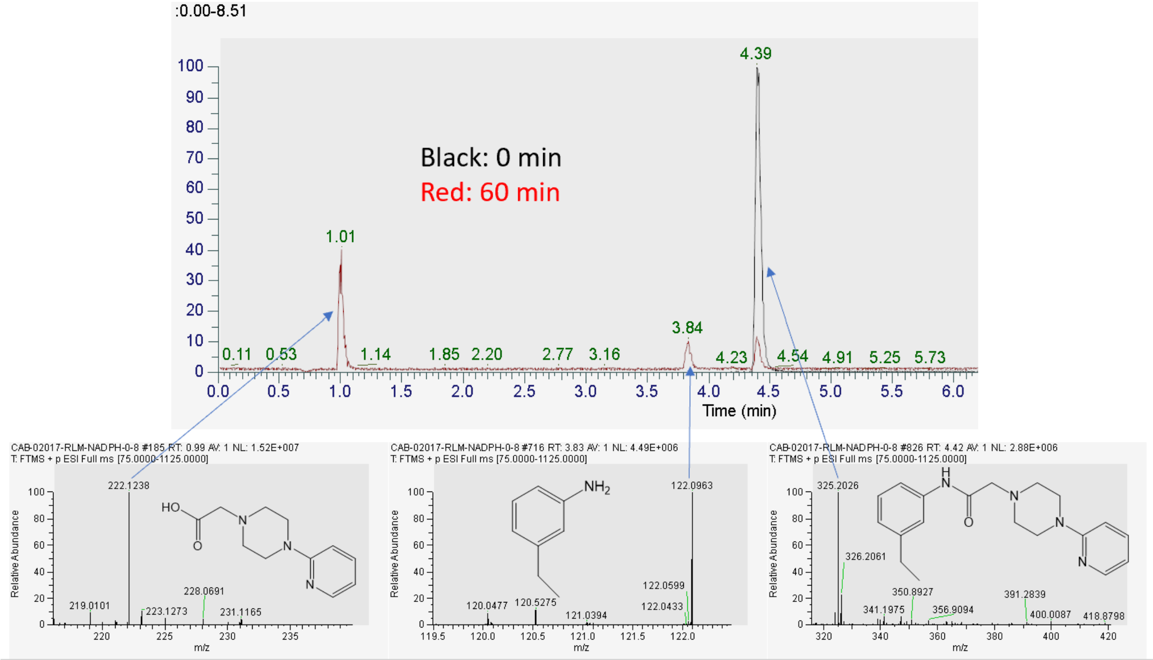

Compound 4 (CAB02-110)

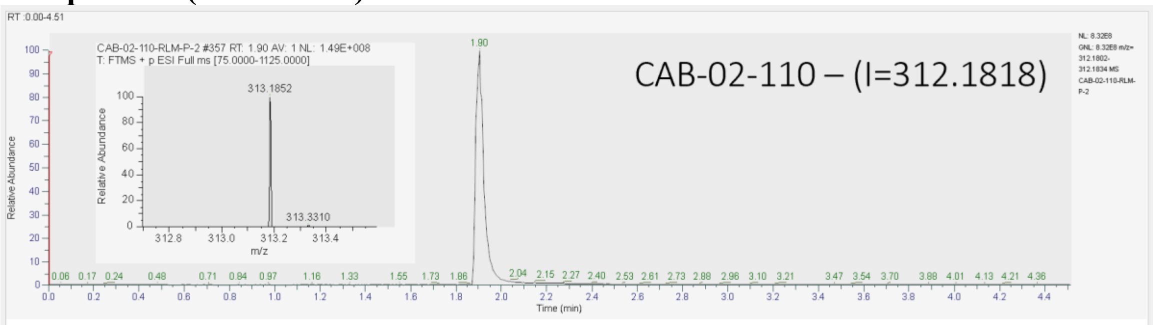

Figure S2. HPLC traces and MS traces of compounds 5-6. Note the metabolite identifications for **5**, and **6**, each showing dealkylated metabolic products with cleavage occurring at the amide linker.

Compound 5 (CAB03-015)

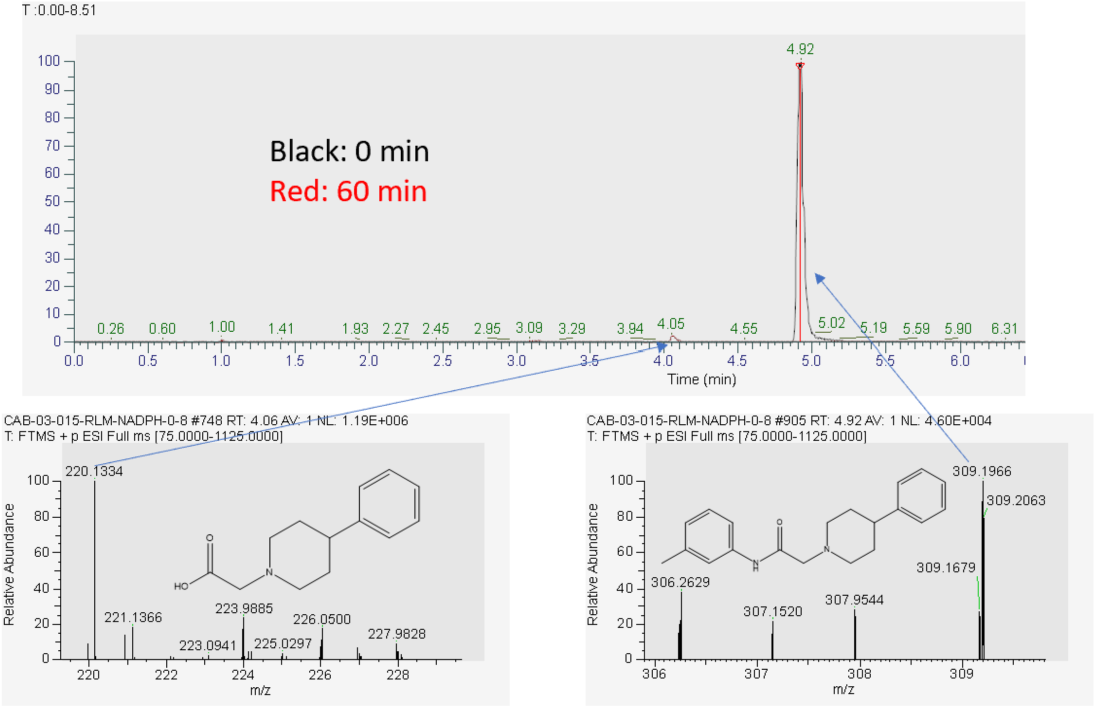

Compound 6 (CAB02-011)

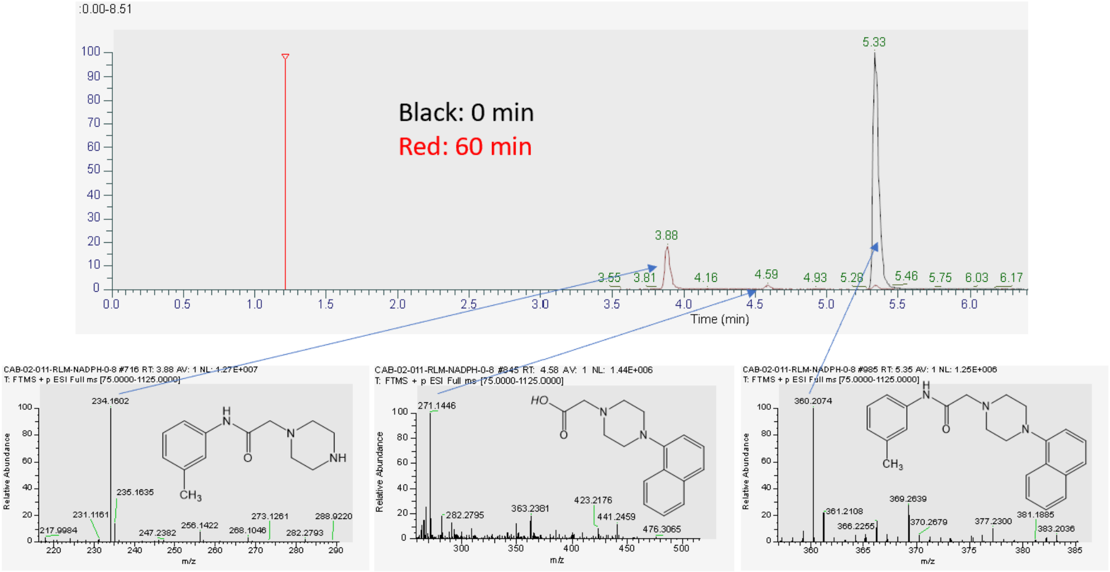

Figure S3. HPLC traces and MS traces of compound 7.

Compound 7 (CAB02-003)

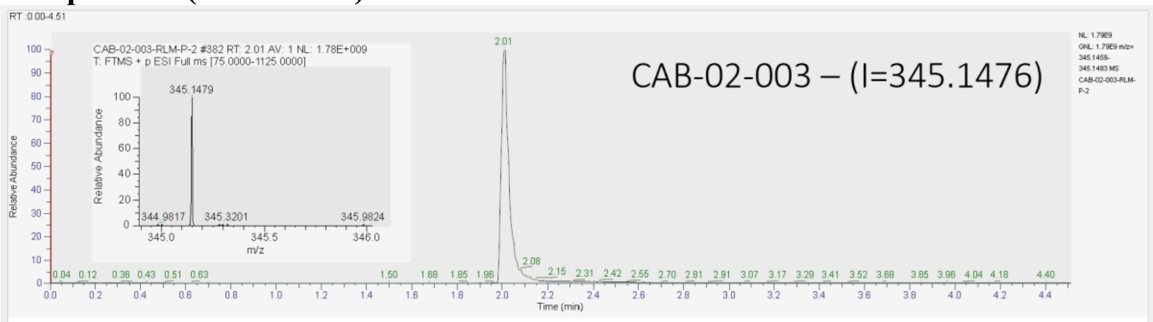

Figure S4. HPLC traces and MS traces of compounds 14-16.

Compound 14 (FMJ-01-045)

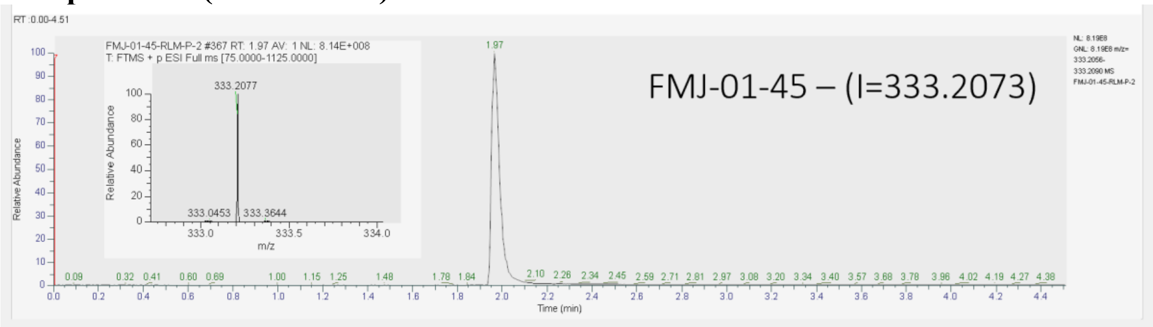

Compound 15 (FMJ-01-038)

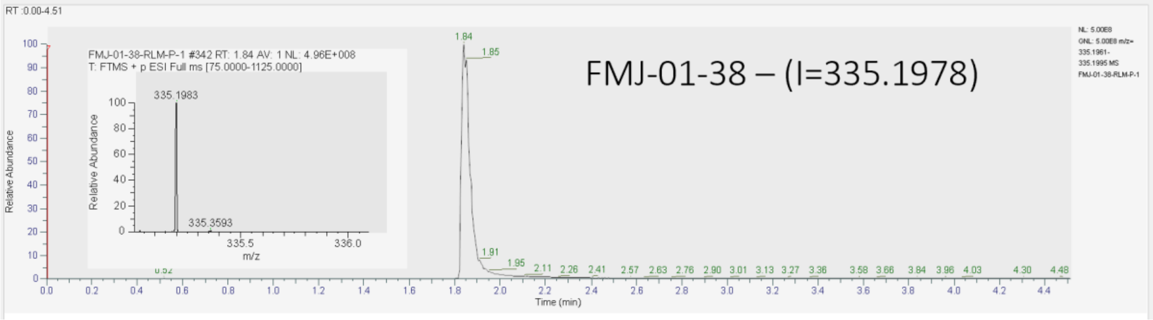

Compound 16 (FMJ-01-053)

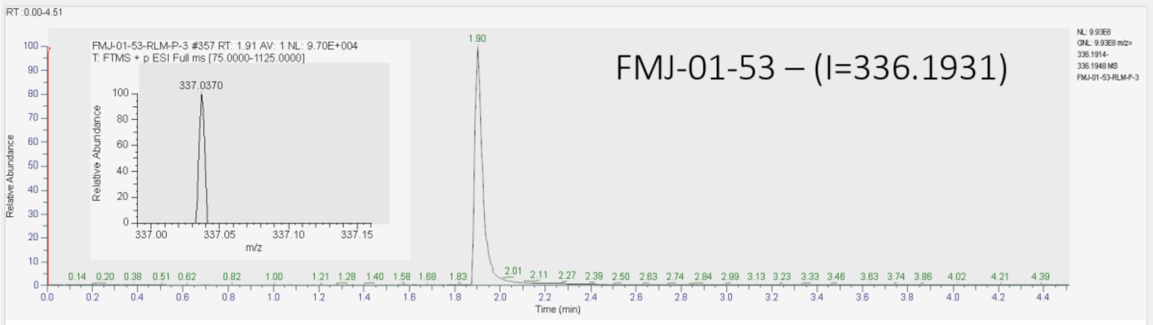

Figure S5. HPLC traces and MS traces of compounds 17-19.

Compound 17 (FMJ-01-054)

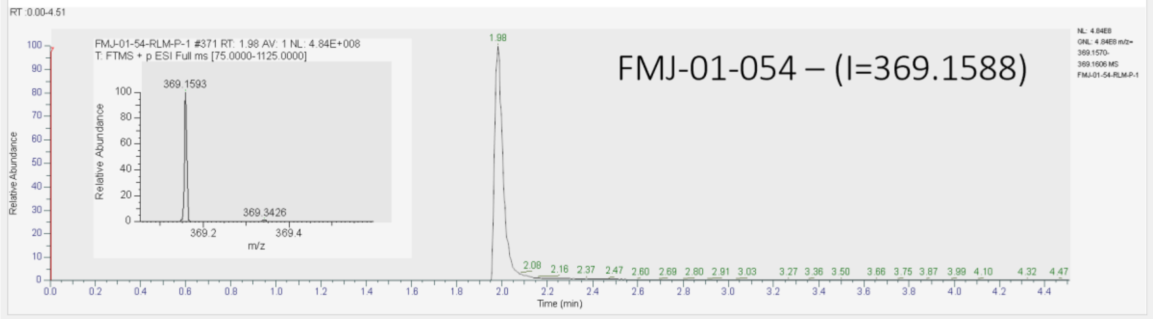

Compound 18 (FMJ-01-042)

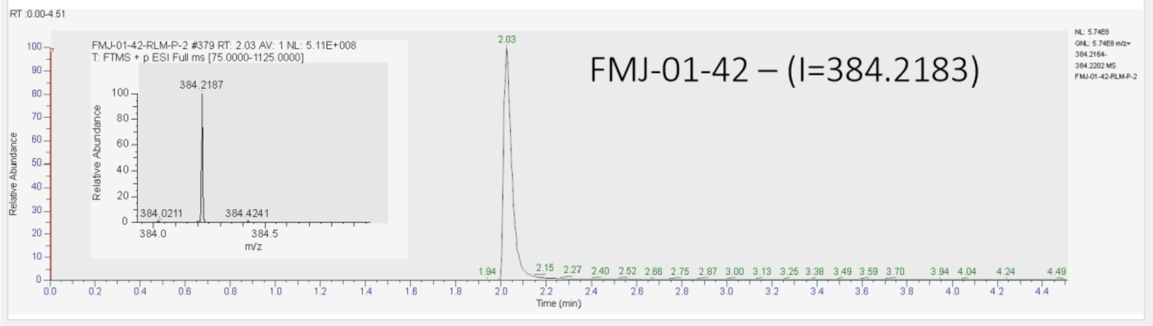

Compound 19 (FMJ-01-044)

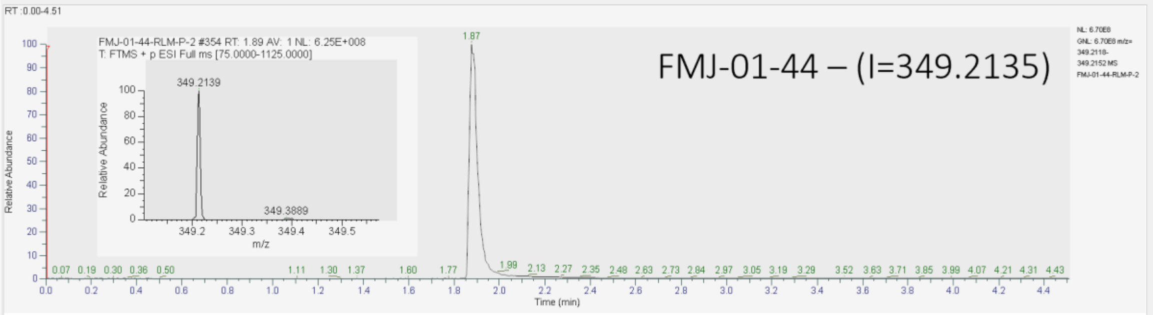

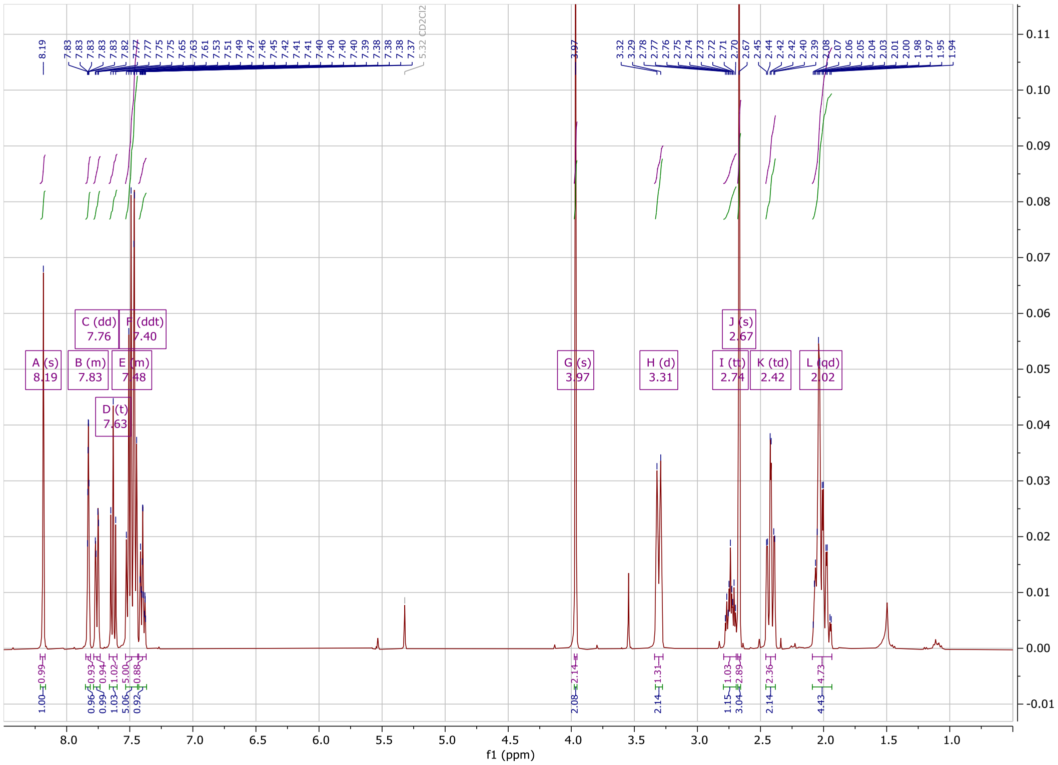

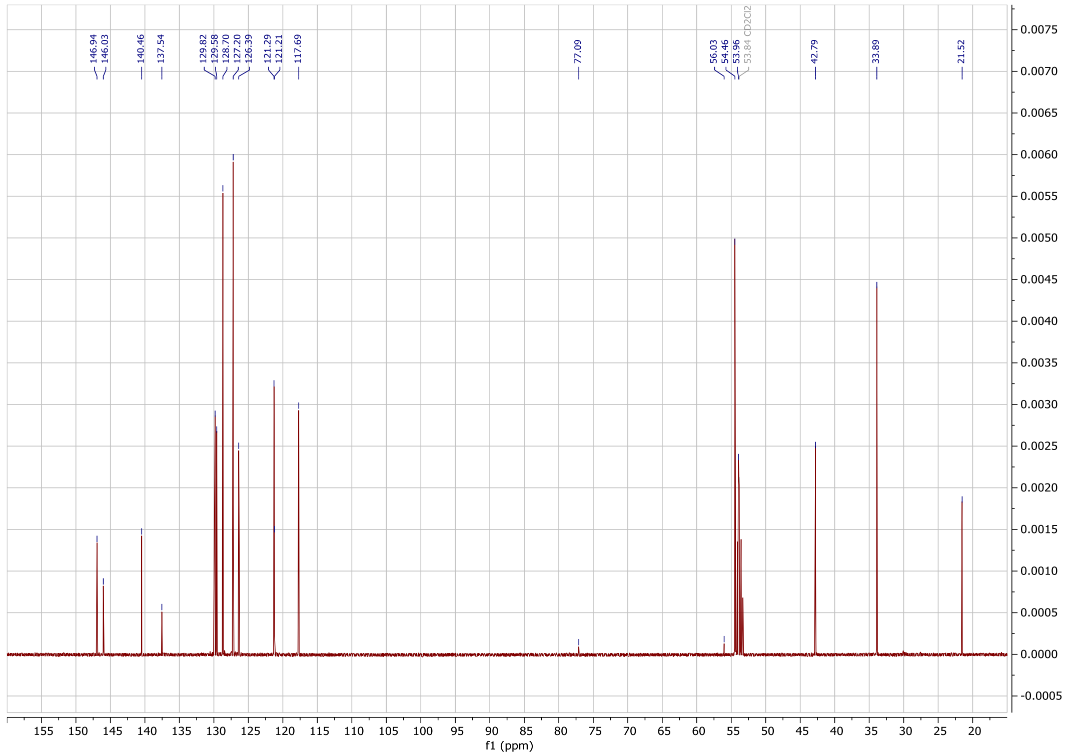

Figure S6. ¹H and ¹³C NMR Spectra for compound 14 (FMJ-01-045)

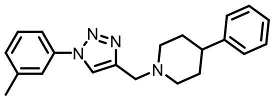

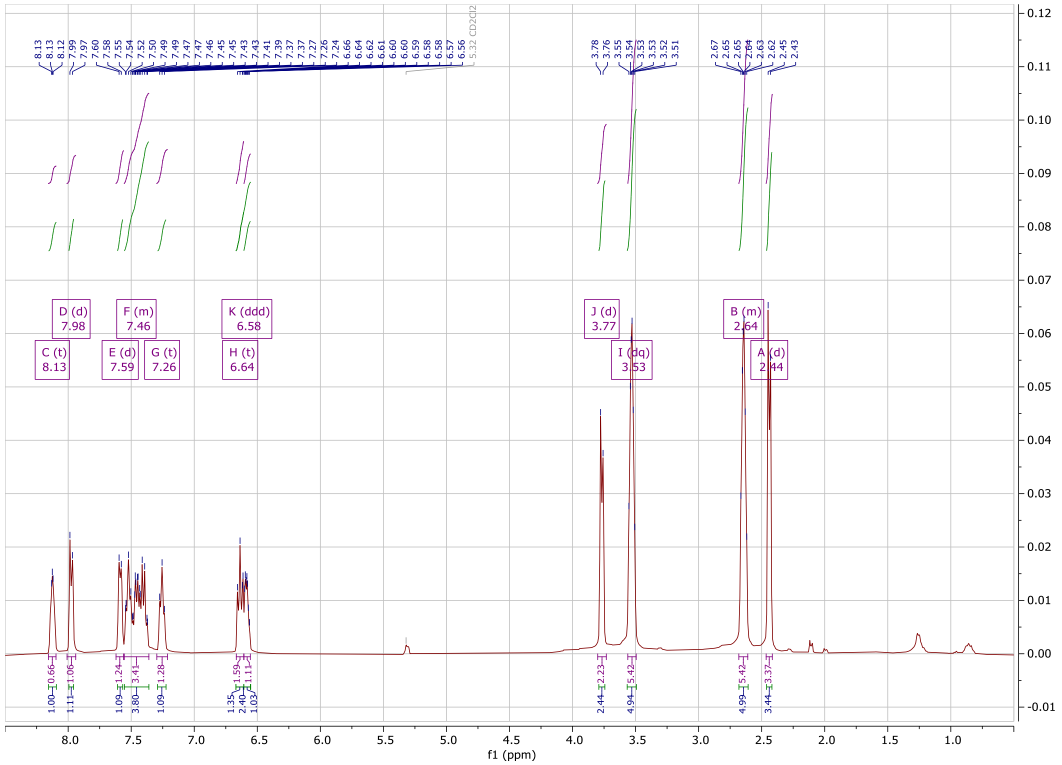

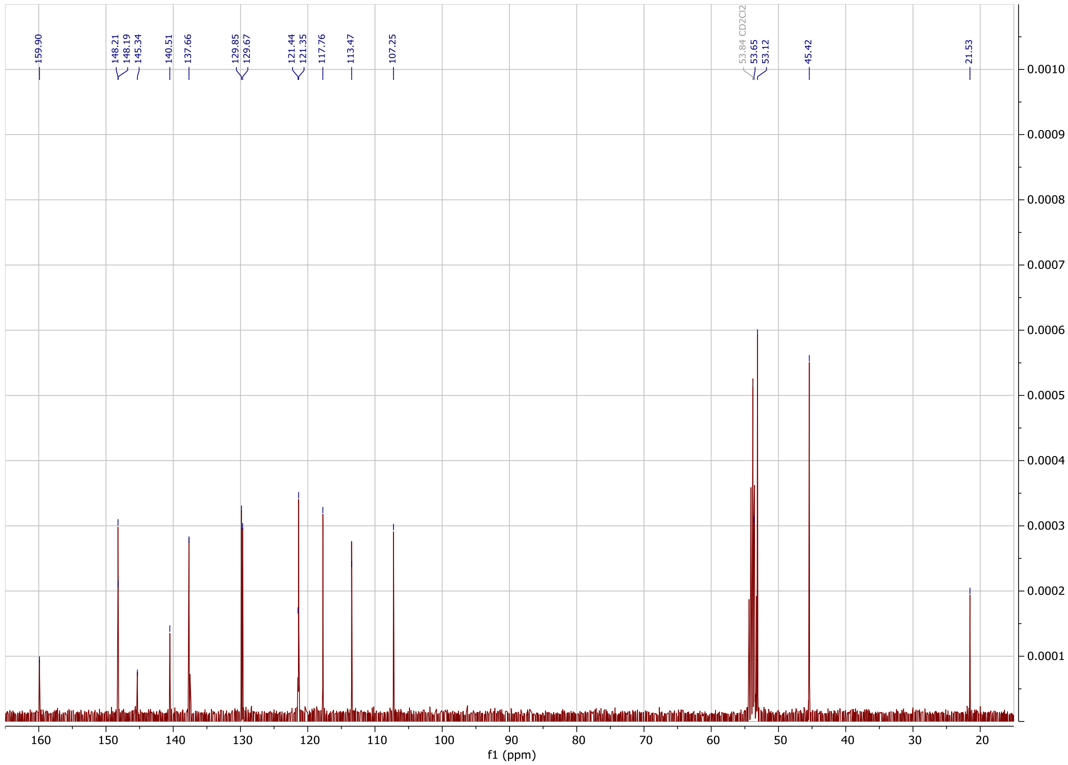

Figure S7. ^{1}H and ^{13}C NMR Spectra for compound 15 (FMJ-01-038)

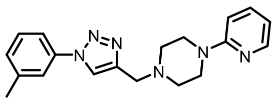

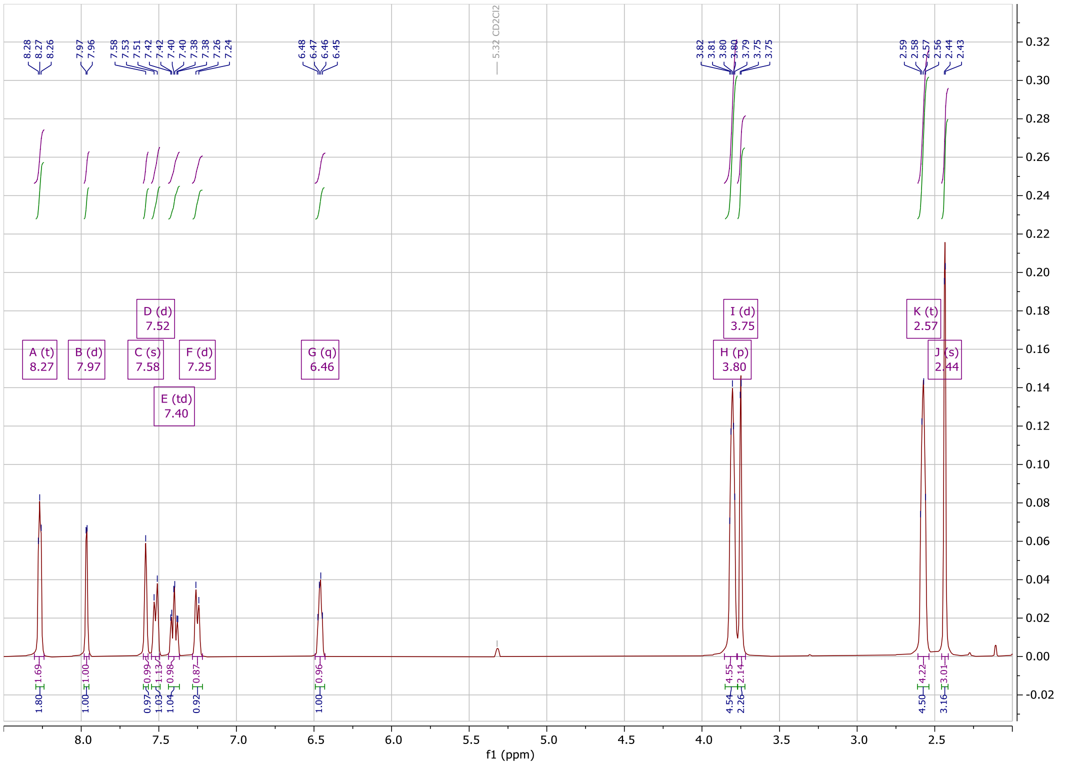

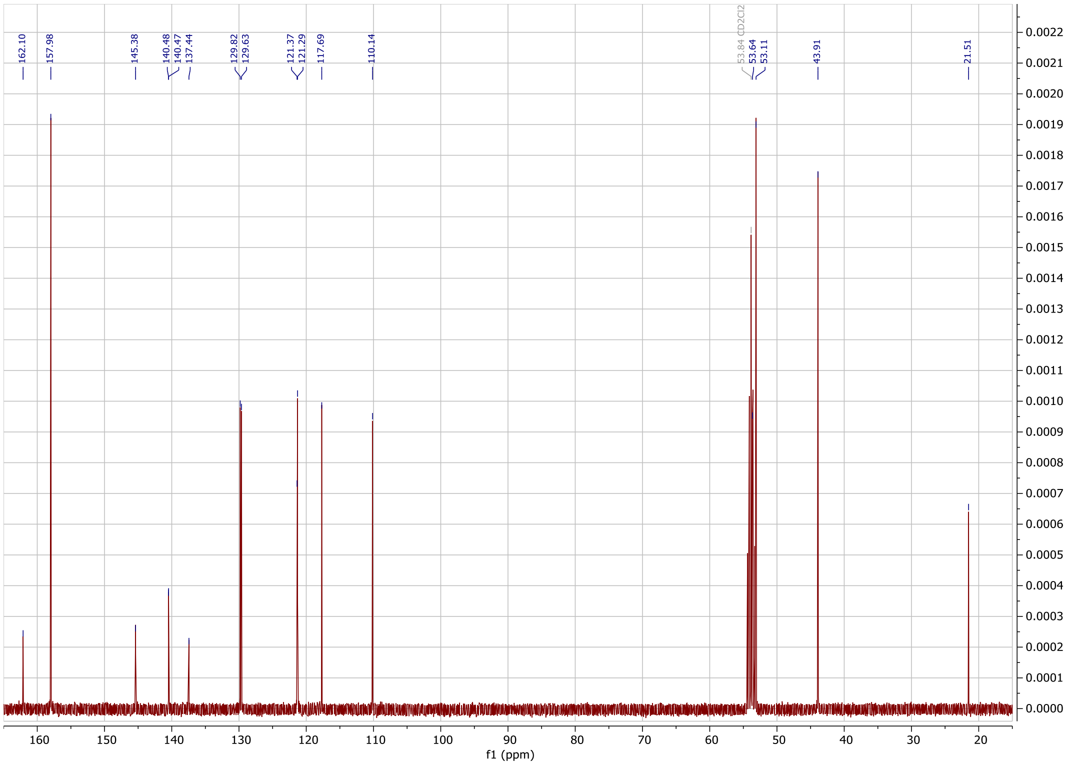

Figure S8. ^{1}H and ^{13}C NMR Spectra for compound 16 (FMJ-01-053)

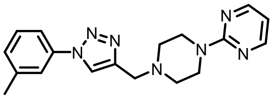

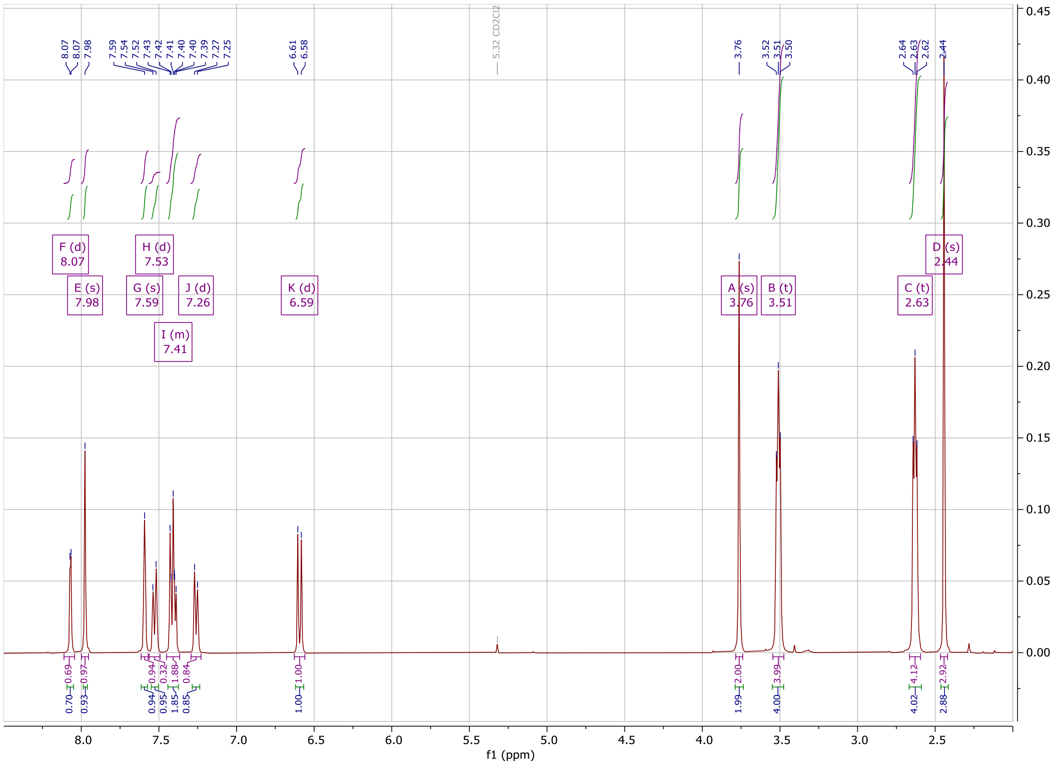

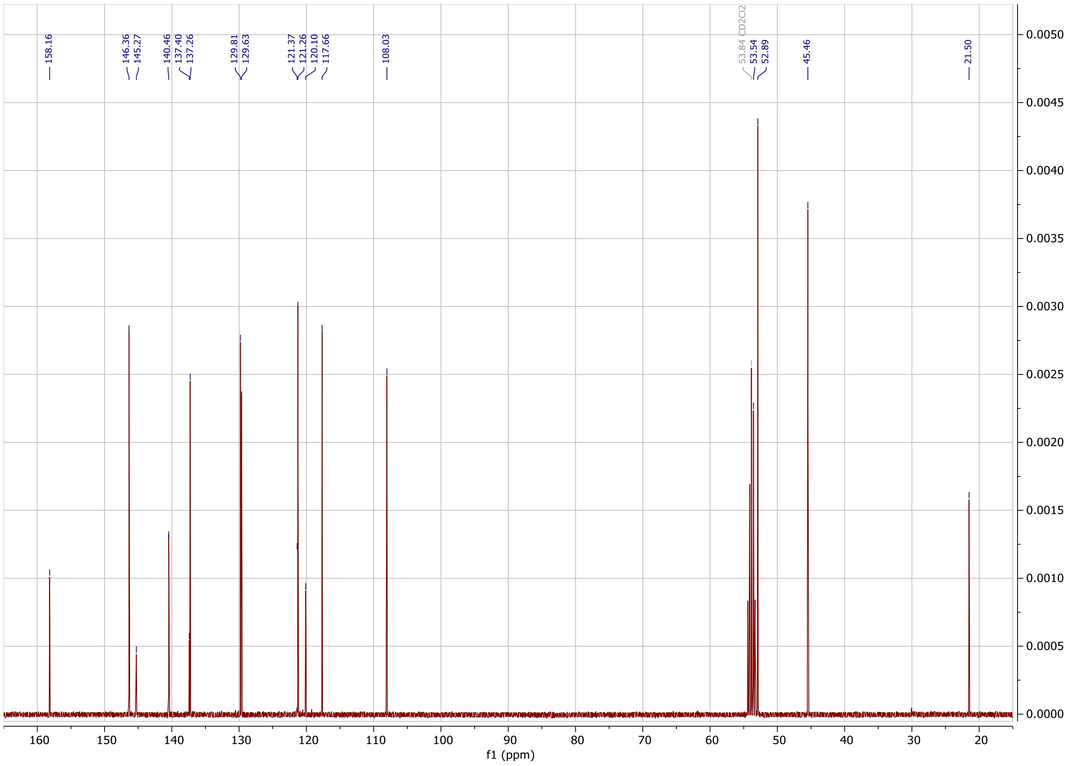

Figure S9. ^{1}H and ^{13}C NMR Spectra for compound 17 (FMJ-01-054)

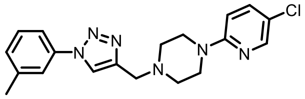

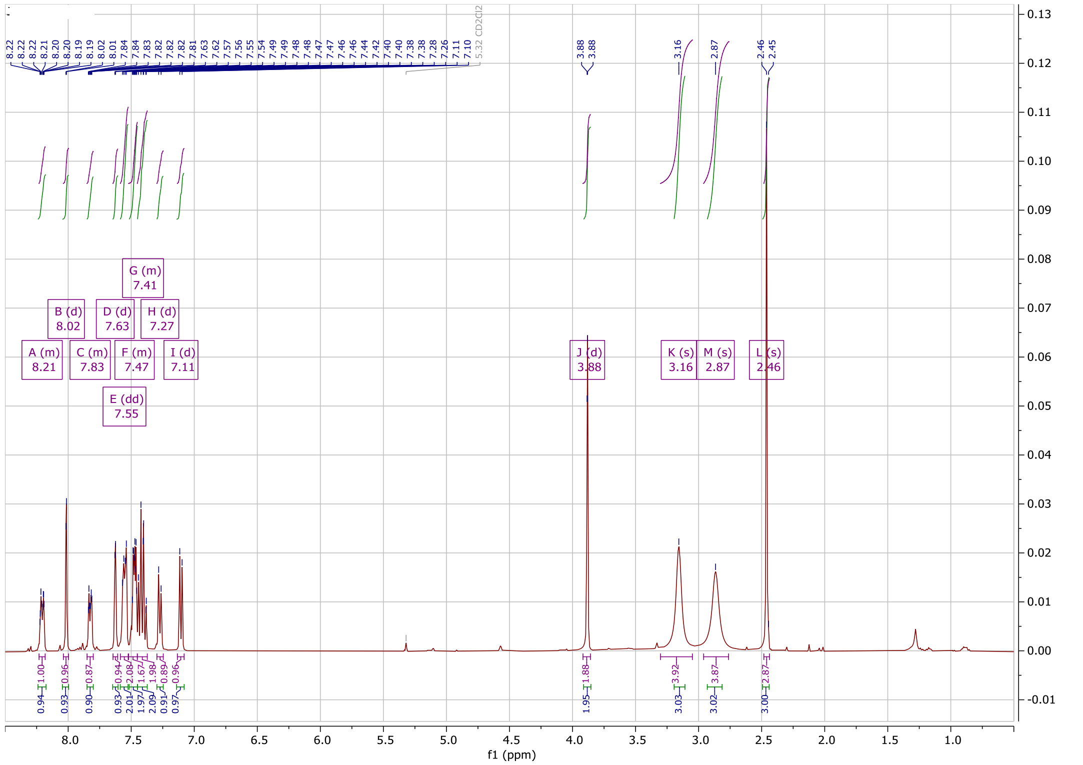

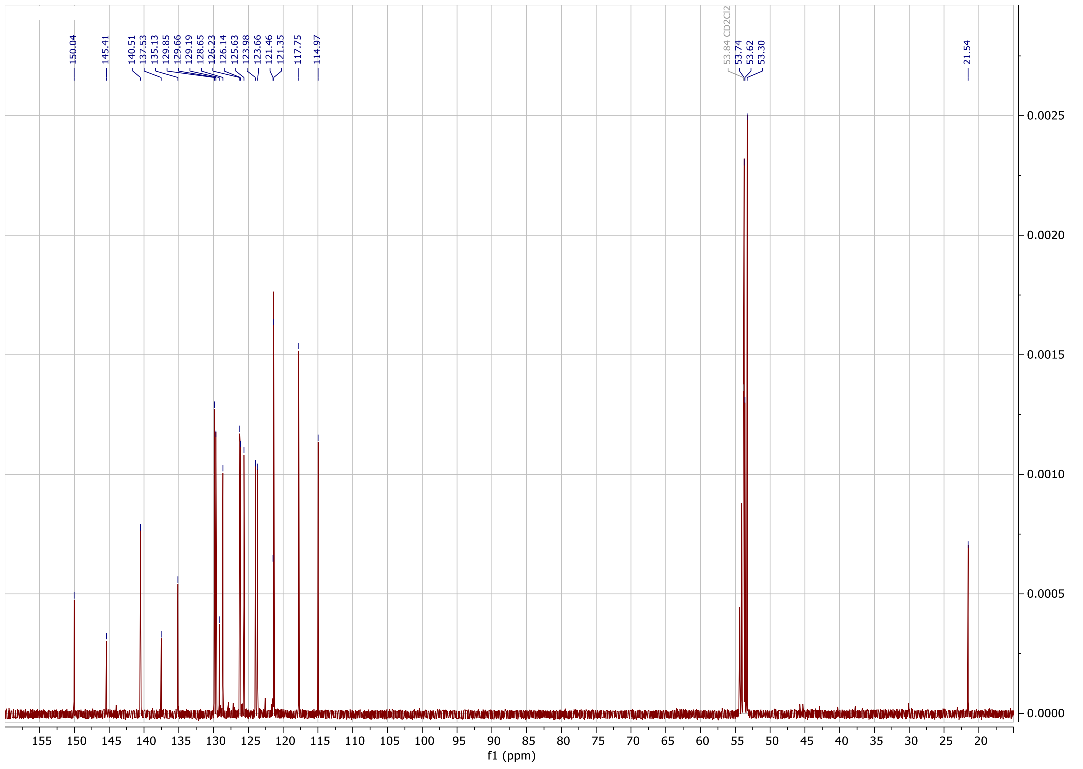

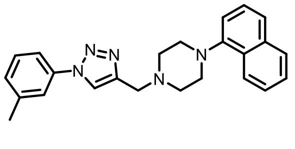

Figure S10. ^{1}H and ^{13}C NMR Spectra for compound 18 (FMJ-01-042)

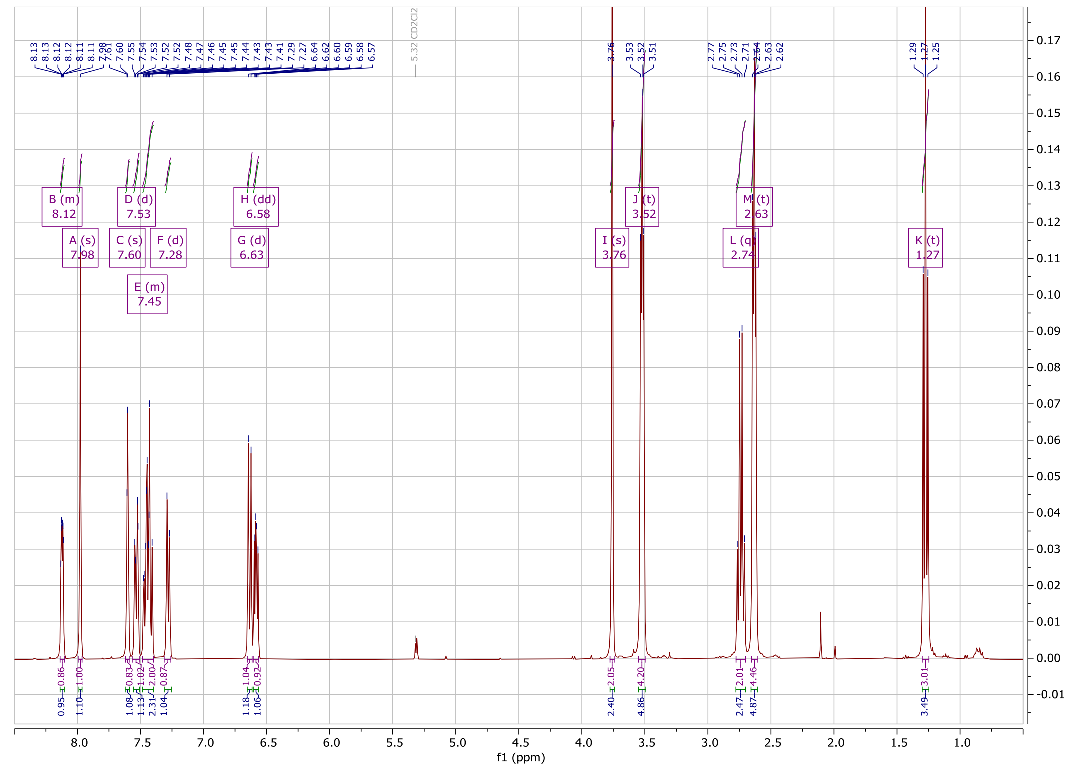

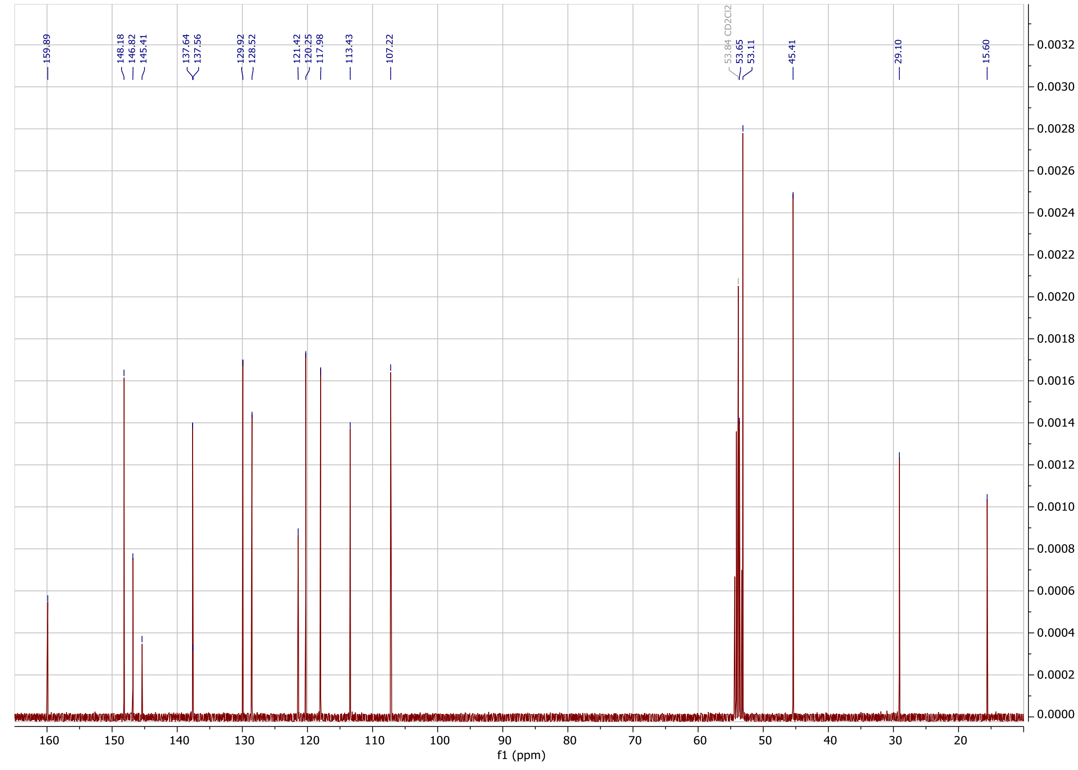

Figure S11. ^{1}H and ^{13}C NMR Spectra for compound 19 (FMJ-01-044)

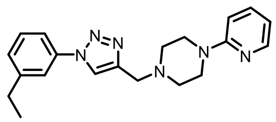

Table S2. Compounds (14, 15, 17, 18) effects on D_1-like dopamine receptors.

Psychoactive Drug Screening Program (PDSP) results from primary and secondary assays on an array of dopamine receptors of selected compounds.

	**15** FMJ-01-038		**14** FMJ-01-045		**17** FMJ-01-054		**18** FMJ-01-042	
**Receptor**	**Primary screen (% inhibition)**	**Secondary assay K_i (nM)^c**	**Primary screen (% inhibition)**	**Secondary assay K_i (nM)^c**	**Primary screen (% inhibition)**	**Secondary assay K_i (nM)^c**	**Primary screen (% inhibition)**	**Secondary assay K_i (nM)^c**
D1	2.16	NT	20.2	NT	16.68	NT	59.3	783
D5	1.76	NT	16.0	NT	6.22	NT	25.7	NT
	Receptors were initially tested with 10 µM selected compounds, and % inhibition measured compared to a known reference compound. Receptors with greater than 50% inhibition were selected for full assays to determine the affinity of compounds **(14, 15, 17, 18)** for the receptor. ^{c} NT – Not Tested due to > 50% inhibition in primary assessment.							

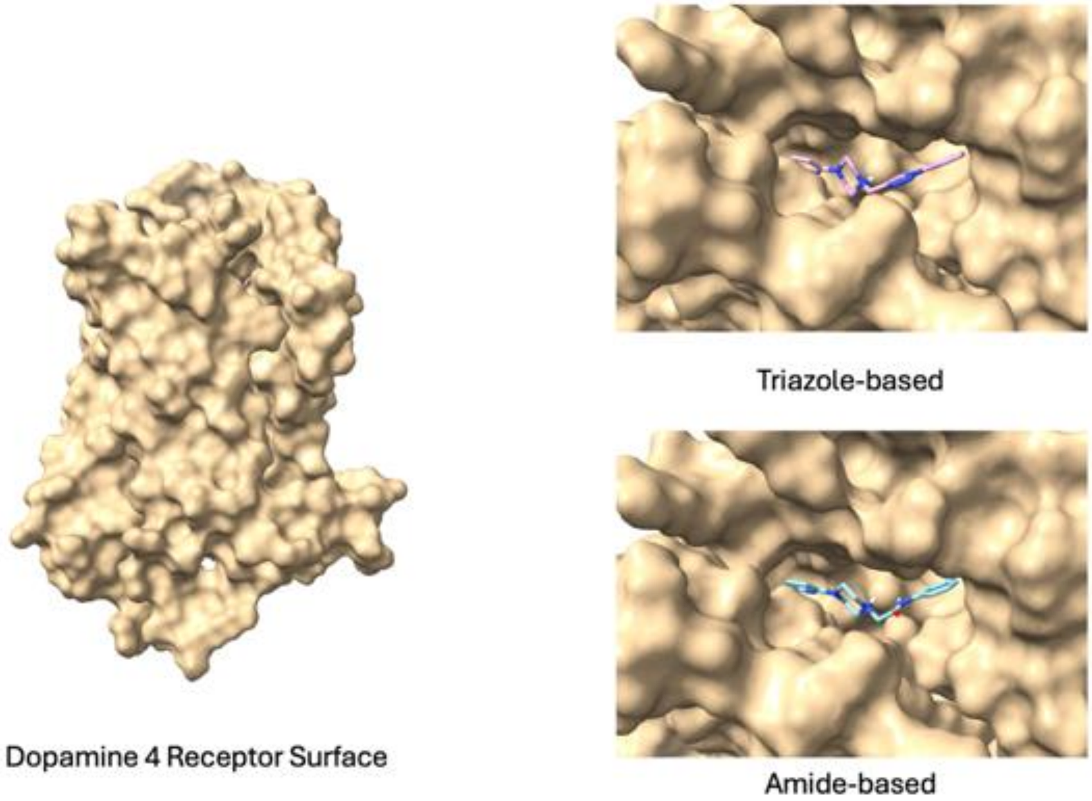

Figure S12. Image of the D_4R surface and aerial view of the binding pocket docked with matching representatives of triazole-based (**17**; top inset) and amide-based (**2**; bottom inset) analog sets.

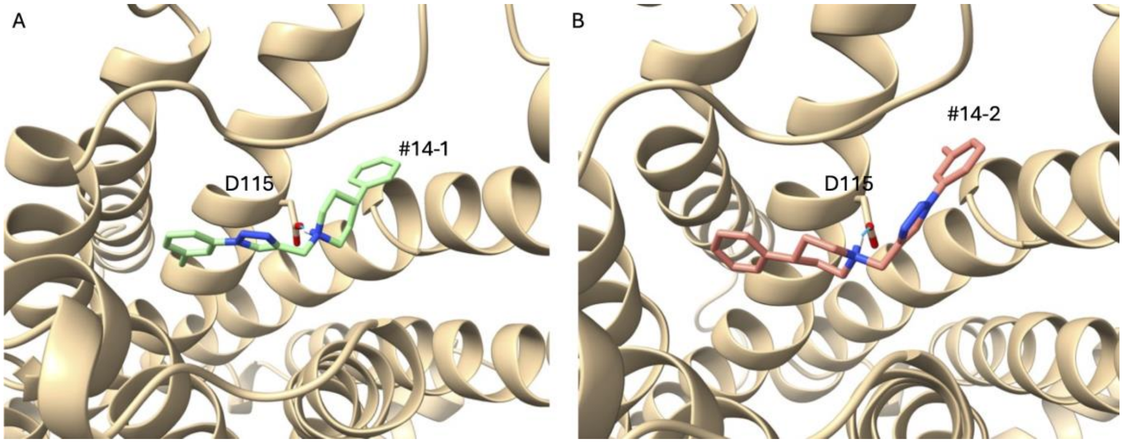

Figure S13. Binding poses for compound **14** in the “opposite pose” (A) and the “consistent pose” (B).

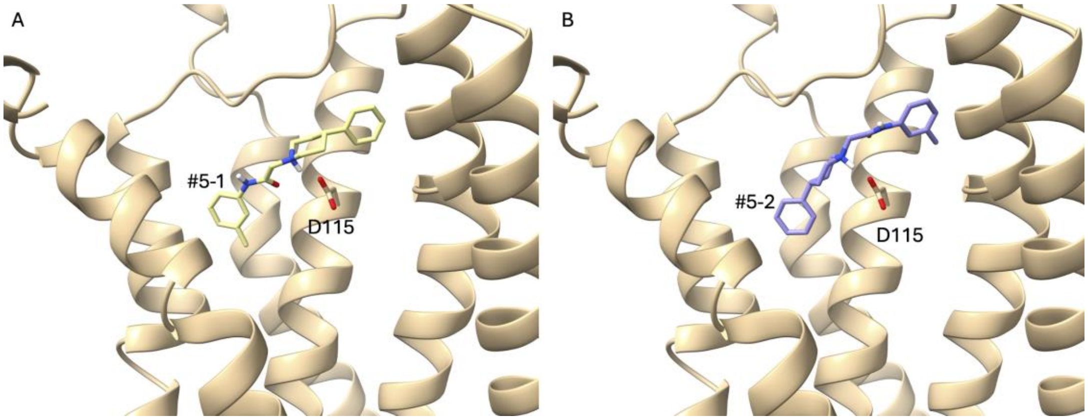

Figure S14. Binding poses for compound **5** in the “opposite pose” (A) and the “consistent pose” (B).
